# Supplementary material for: Inducing mismatch repair deficiency sensitizes immune-cold neuroblastoma to anti-CTLA4 and generates broad anti-tumor immune memory
Source: Mol Ther. 2022 Sep 6;31(2):535–51. doi: 10.1016/j.ymthe.2022.08.025 (PMC9931548; doi:10.1016/j.ymthe.2022.08.025)
Supplement: Document S1. Figures S1–S7 [file mmc1.pdf]

YMTHE, Volume *31*

Supplemental Information

Inducing mismatch repair deficiency sensitizes immune-cold neuroblastoma to anti-CTLA4 and generates broad anti-tumor immune memory

Mikal El-Hajjar, Lara Gerhardt, Megan M Y Hong, Mithunah Krishnamoorthy, Rene Figueredo, Xiufen Zheng, James Koropatnick, and Saman Maleki Vareki

Supplementary Data

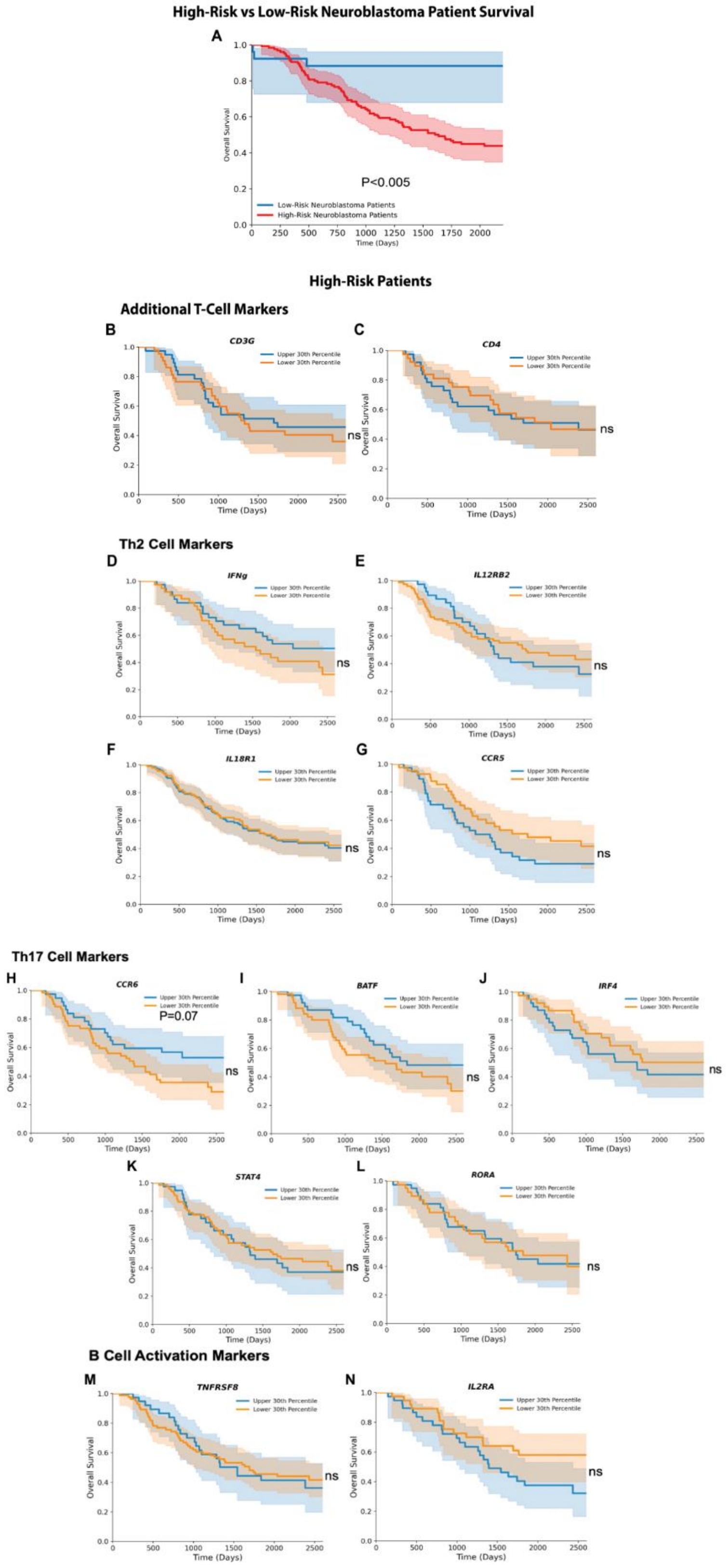

Figure S1: Th1, Th2, Th17 and B-cell gene expression are not associated with improved survival in high-risk neuroblastoma patients. Kaplan-Meier survival analysis by log-rank test of low-risk neuroblastoma patients (n=26) vs. high-risk neuroblastoma patients (n=127) (**A**). Kaplan-Meier survival analysis by log-rank test of high-risk neuroblastoma patients stratified according to upper and lower 30th percentile gene expression of CD3G (**B**) (n=38, n=43), CD4 (**C**) (n=38, n=38), IFNg (**D**) (n=38, n=38), IL12RB2 (**E**) (n=38, n=73), IL18R1 (**F**) (n=127, n=91), CCR5 (**G**) (n=38, n=42), CCR6 (**H**) (n=38, n=62), BATF (**I**) (n=38, n=52), IRF4 (**J**) (n=38, n=38), STAT4 (**K**) (n=38, n=67), RORA (**L**) (n=38, n=38), TNFRSF8 (**M**) (n=38, n=84), IL2RA (**N**) (n=38, n=38). * $p \leq 0.05$, ns (not significant).

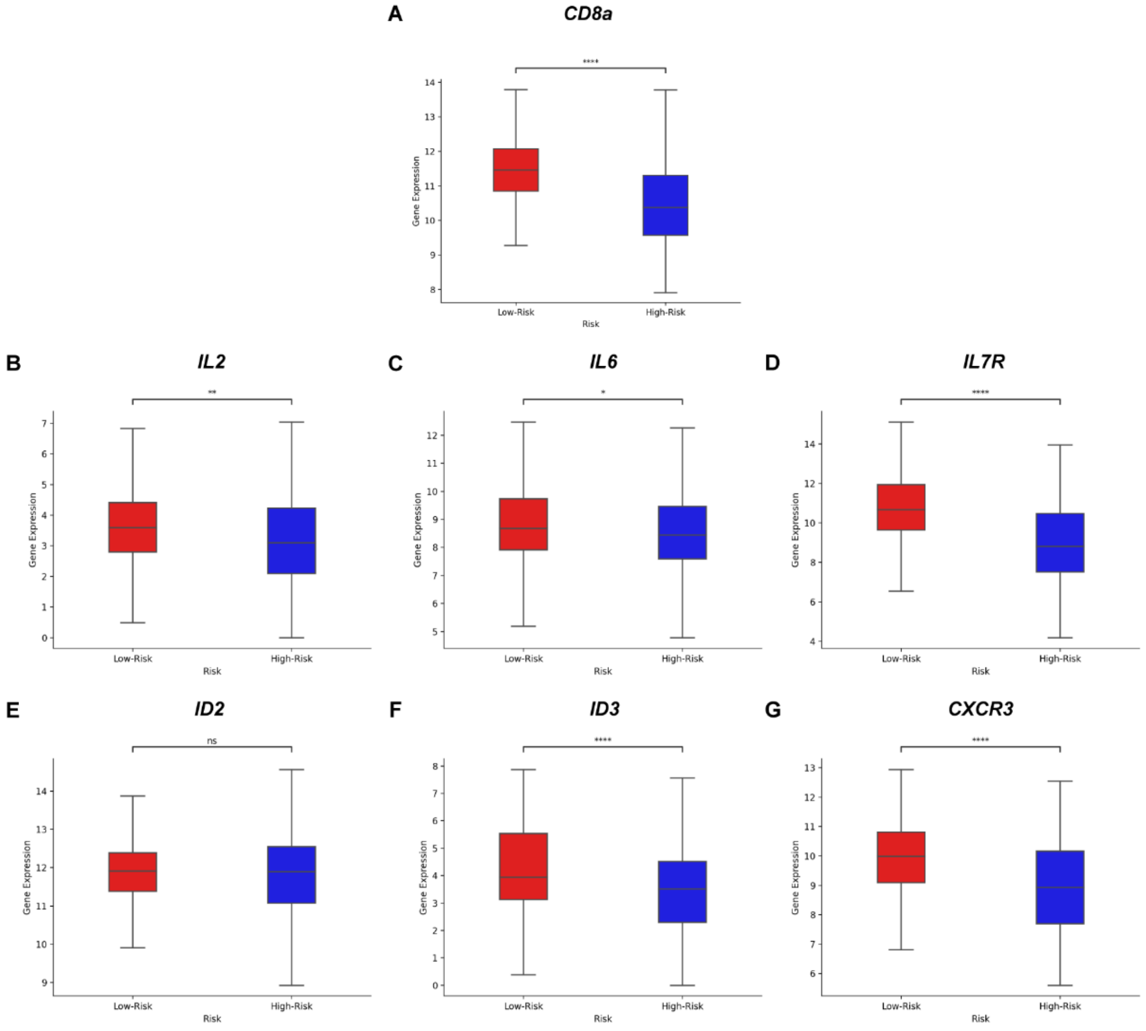

Figure S2: Low-risk neuroblastoma patients have an increased expression of pro-inflammatory genes compared to high-risk neuroblastoma patients. Tumor samples obtained from the neuroblastoma GEO49710 dataset were grouped into low-risk and high-risk subsets respectively (n=322, n=176). Normalized microarray gene expression reads for T-cell associated genes CD3a (**A**), IL2 (**B**), IL6 (**C**), IL7R (**D**), ID2 (**E**), ID3 (**F**) was compared. Statistical analysis was performed by non-parametric Mann-Whitney U test. * $p \leq 0.05$, ** $p \leq 0.01$, *** $p \leq 0.001$, **** $p \leq 0.0001$, ns (not significant).

***MYCN* amplified**

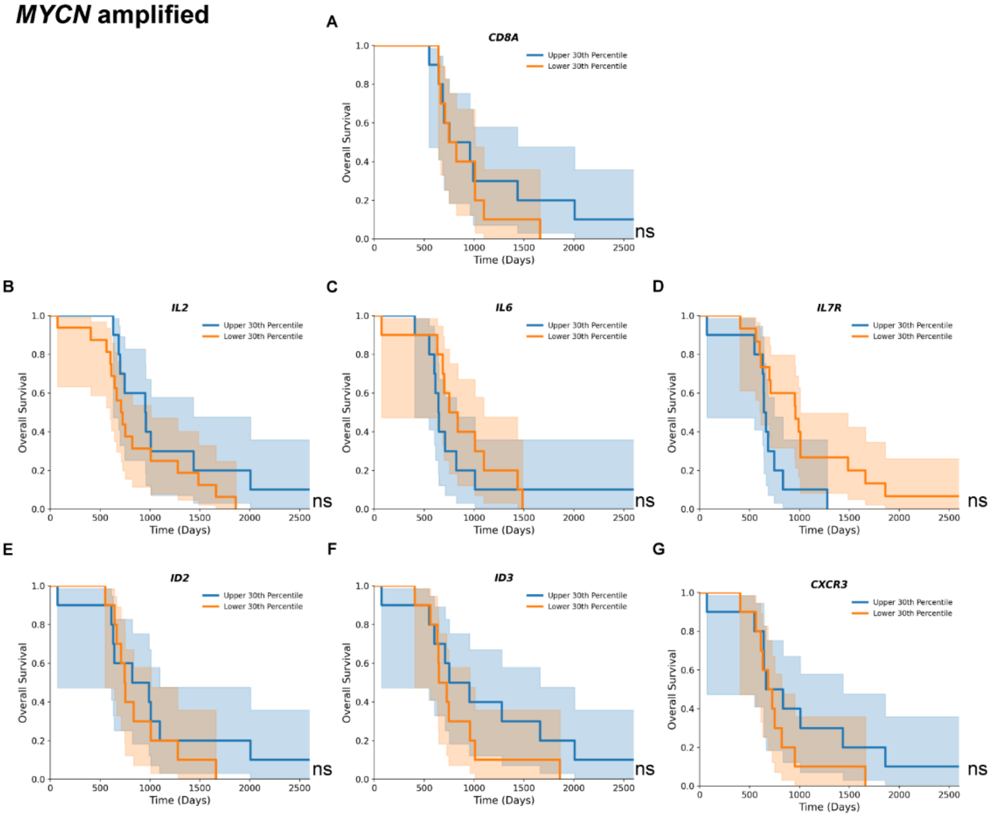

***MYCN* non-amplified**

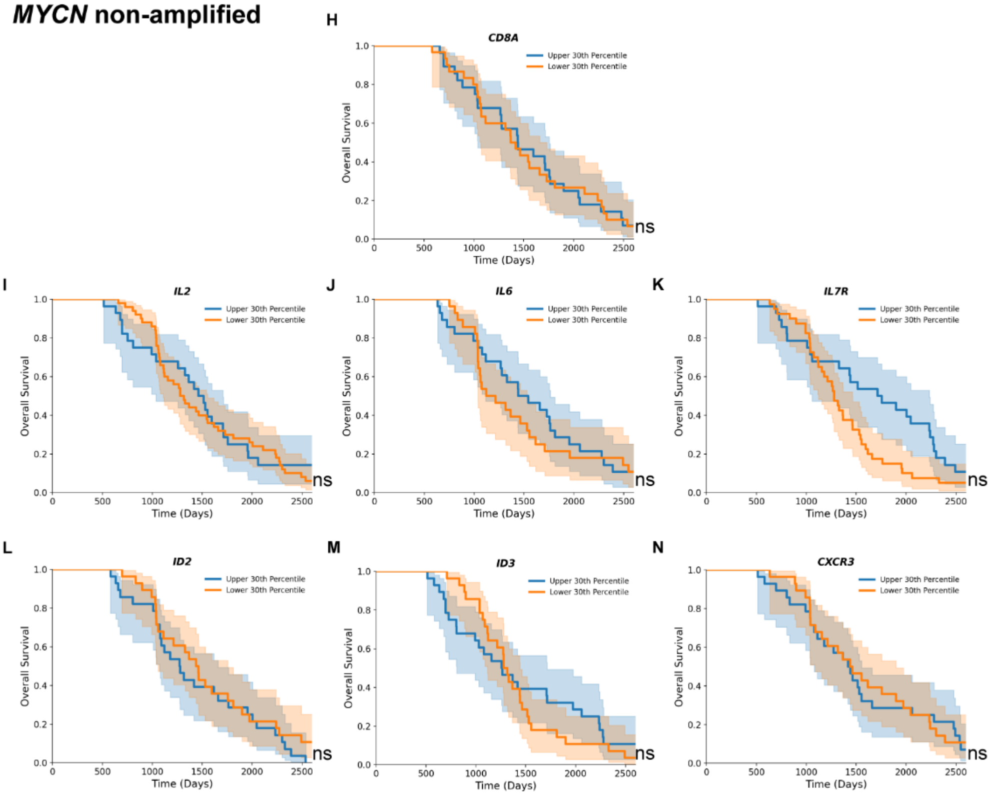

Figure S3: Expression of proinflammatory genes are not correlated with *MYCN* amplification status in high-risk neuroblastoma patients. Kaplan-Meier survival analysis by log-rank test of neuroblastoma patients with *MYCN* amplification stratified according to upper and lower 30th percentile gene expression of CD8a (**A**) (n=10, n=10), IL2 (**B**) (n=10, n=16), IL6 (**C**) (n=10, n=10), IL7R (**D**) (n=10, n=15), ID2 (**E**) (n=10, n=10), ID3 (**F**) (n=10, n=10) and CXCR3 (**G**) (n=10, n=10). Similar analysis was conducted in non-amplified neuroblastoma patients, stratifying patients according to expression of genes CD8a (**H**) (n=28, n=30), IL2 (**I**) (n=28, n=50), IL6 (**J**) (n=28, n=28), IL7R (**K**) (n=28, n=40), ID2 (**L**) (n=28, n=28), ID3 (**M**) (n=28, n=28) and CXCR3 (**N**) (n=28, n=28). ns (not significant)

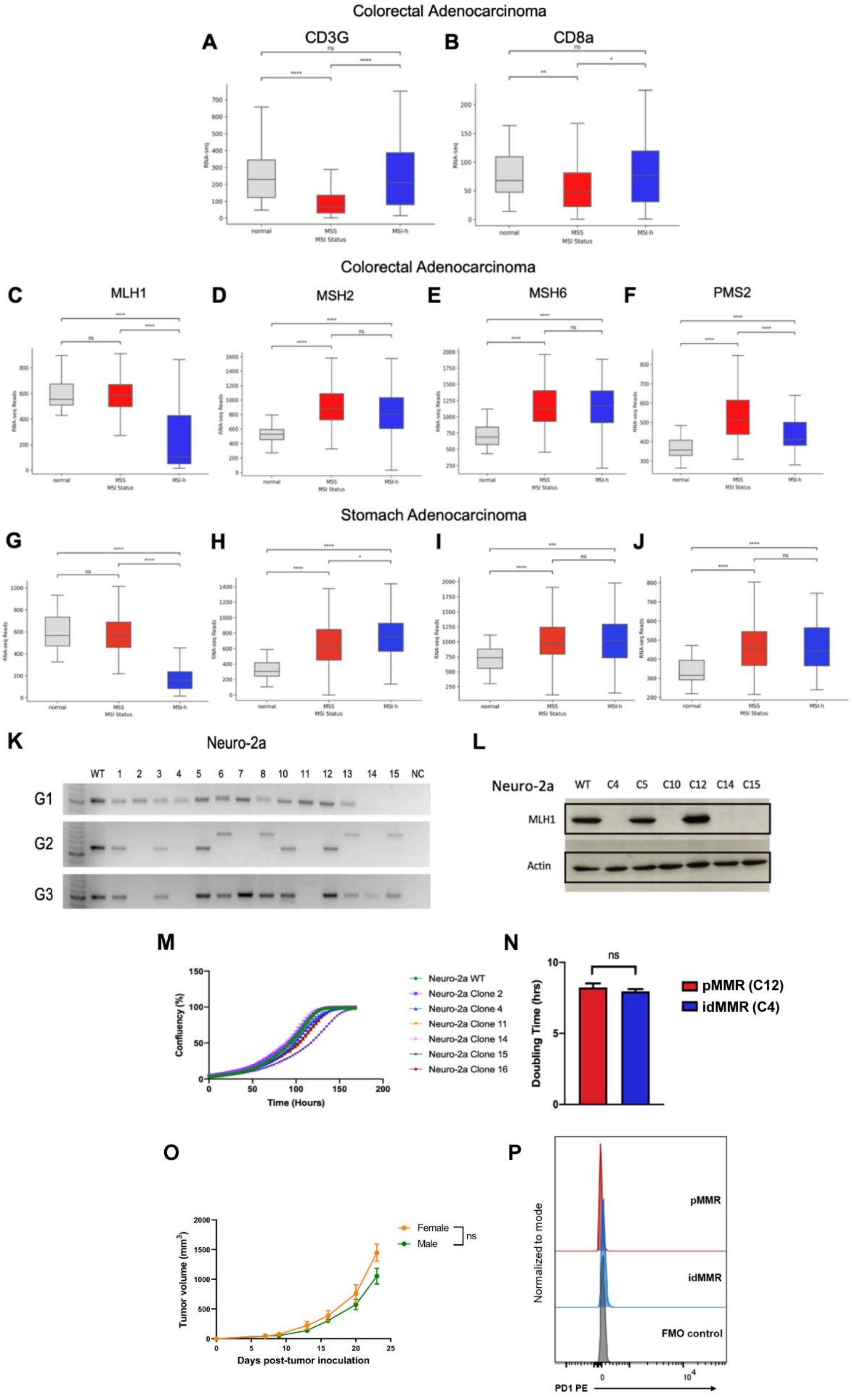

Colorectal Adenocarcinoma
A
CD3G
B
CD8a
RNA-seq
normal
MSS
MSI-h
MSI Status
Colorectal Adenocarcinoma
C
MLH1
D
MSH2
E
MSH6
F
PMS2
RNA-seq Reads
Stomach Adenocarcinoma
G
H
I
J
K
Neuro-2a
WT 1 2 3 4 5 6 7 8 10 11 12 13 14 15 NC
G1
G2
G3
L
Neuro-2a
WT C4 C5 C10 C12 C14 C15
MLH1
Actin
M
Confluency (%)
Time (Hours)
Neuro-2a WT
Neuro-2a Clone 2
Neuro-2a Clone 4
Neuro-2a Clone 11
Neuro-2a Clone 14
Neuro-2a Clone 15
Neuro-2a Clone 16
N
ns
Doubling Time (hrs)
pMMR (C12)
idMMR (C4)
O
Tumor volume (mm3)
Days post-tumor inoculation
Female
Male
ns
P
Normalized to mode
pMMR
idMMR
FMO control
PD1 PE

Figure S4: MMR deficiency enhances T-cell based immune surveillance in tumors. Normalized RNA-seq data for T-cell genes *CD3G* (**A**), *CD8A* (**B**), *MLH1* (**C**), *MSH2* (**D**), *MSH6* (**E**), *PMS2* (**F**) from the TCGA database colorectal adenocarcinoma (COAD READ) dataset and *MLH1* (**G**), *MSH2* (**H**), *MSH6* (**I**), *PMS2* (**J**) from the stomach adenocarcinoma (STAD) dataset. COAD READ and STAD samples were grouped into normal, MSS and MSI-h tissues (n=51, n=257, n=51), (n=35, n=276, n=80) respectively. Statistical analysis was performed by non-parametric Mann-Whitney U test. * $p \leq 0.05$, ** $p \leq 0.01$, *** $p \leq 0.001$, **** $p \leq 0.0001$, ns (not significant). Neuro-2a cells were transfected with *MLH1* CRISPR CAS9 knockout plasmid. PCR results for clones resulted from transfection of neuro-2a cells with *MLH1* CRISPR/Cas9 plasmid (top to bottom: amplification with guide 1 (G1), G2 and G3 primers respectively), WT represents amplification of non-transfected wild type (WT) Neuro-2a cells as a positive control for each primer and NC represents a negative control for each set of PCR products (no template) (**K**). Expression of MLH1 in the parental cells and several putative knockout clones was analyzed by Western Blot(**L**). WT neuro-2a and *MLH1* KO neuro-2a cell growth profiles over a 90-hour period imaged using Incucyte (**M**). WT neuro-2a cells (pMMR) (n=9) and *MLH1* KO neuro-2a cells (idMMR) (n=9) were seeded on day 0 and counted on day 1 and were counted again on day 5 to analyze the fold increase of each neuro-2a clone (**N**). Results are representative of 3 pooled experiments. Comparison of idMMR tumor growth in immunocompetent A/J male and female mice (n=5, n=5) (**O**). Flow cytometric analysis of PD1 expression on 8-week pMMR and idMMR neuro-2a cells (**N**). Statistical analysis was performed by unpaired two-tailed Student's t-test, ns (not significant).

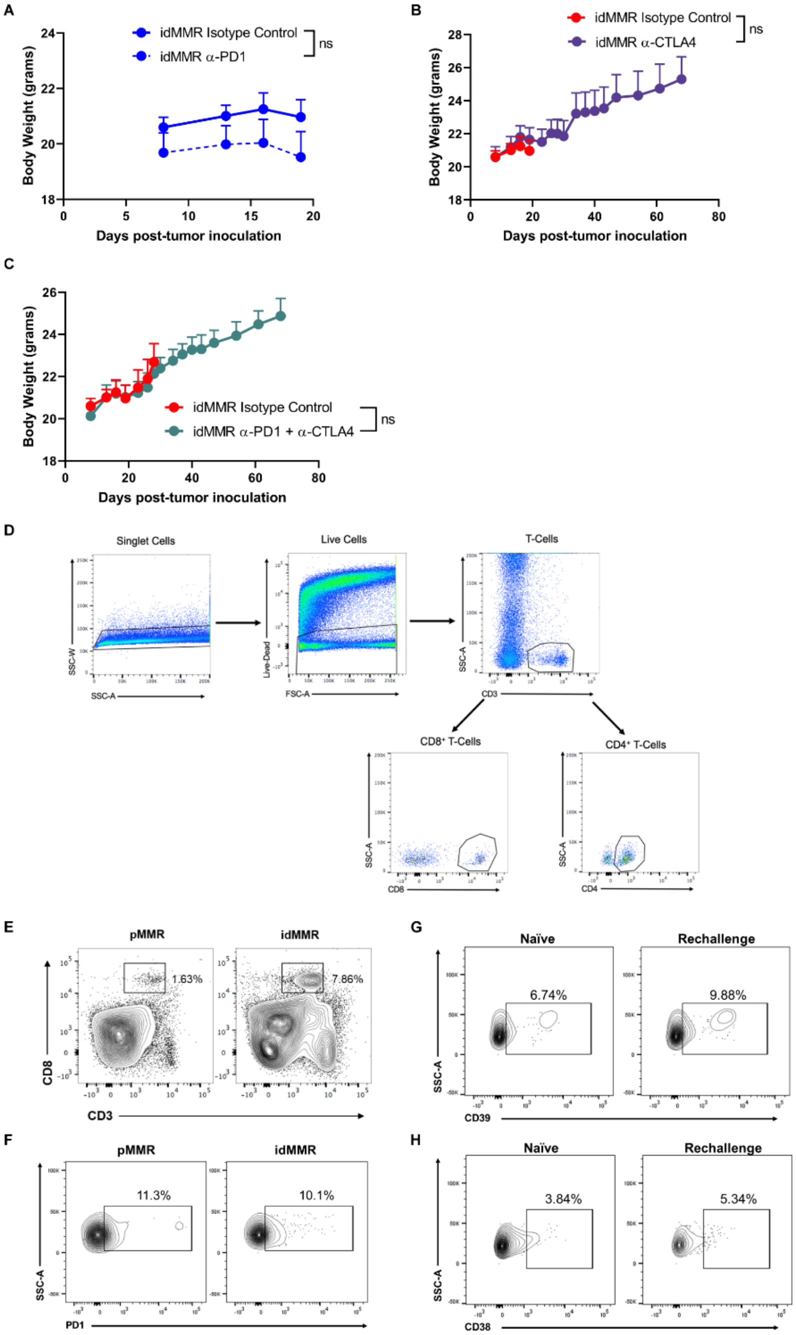

Figure S5: Weight of mice throughout anti-PD1 and anti-CTLA4 treatment. Animals were treated with isotype control antibodies or anti-PD1 antibody (**A**), anti-CTLA4 (**B**), or combination of anti-PD1 and anti-CTLA4 antibodies and their body weight was measured overtime (**C**). Representative flow cytometry gating strategy. Singlet cells were gated and doublet and triplet are excluded. Dead cells were excluded using Zombie NIR live-dead dye. Live T-cells were gated using CD3 marker. CD8 and CD4 T-cells were gated from the T-cell population using CD8 and CD4 markers. Gates were determined using a fluorescence minus one (FMO) control (**D**). Representative plots of $CD3^+CD8^+$ (**E**) and $PD1^+$ $CD8^+$ T-cells (**F**). Representative plots of $CD39^+$ $CD8^+$ T-cells (**G**) and $CD38^+$ $CD4^+$ T-cells (**H**).

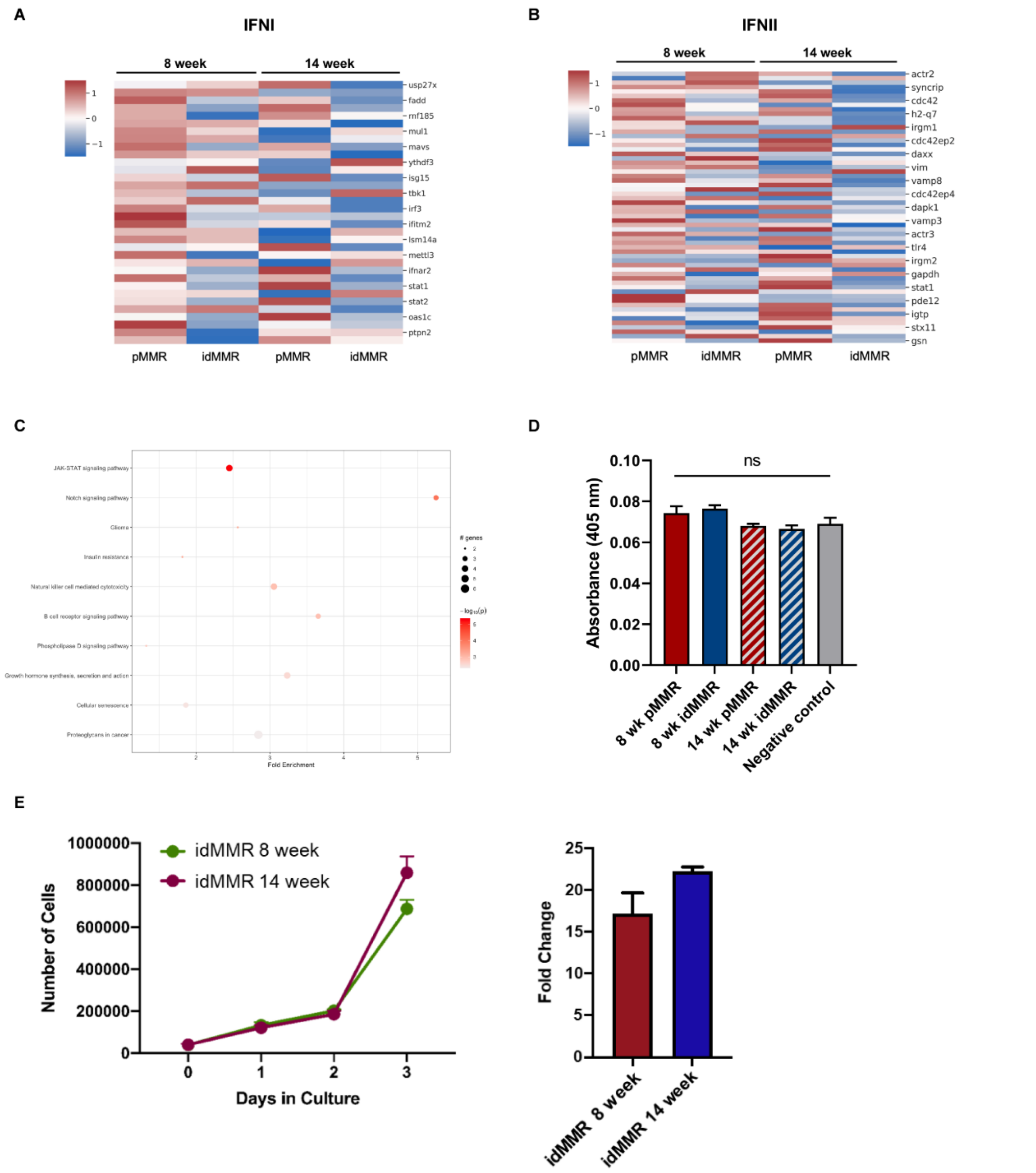

Figure S6: IFN-related gene expression of pMMR and idMMR neuroblastoma cells at 8 weeks and 14 weeks of culture and growth kinetics. Heat maps of the expression of IFNI- and IFNII-related genes in pMMR and idMMR neuroblastoma cells (**A**, **B**). Pathway enrichment analysis of downregulated pathways in idMMR cells compared to pMMR cells at 8 weeks (**C**). IFN-β production from cell culture supernatant measured by ELISA (**D**). Comparison of growth kinetics of idMMR neuroblastoma cells at 8 and 14 weeks of culture. 8-week and 14-week idMMR cells were seeded on day 0 and counted on day 1 and 3 to analyze the cell growth over time fold. Fold increase of both cell lines grown was calculated using cell counts on day 1 and 3 (**E**).

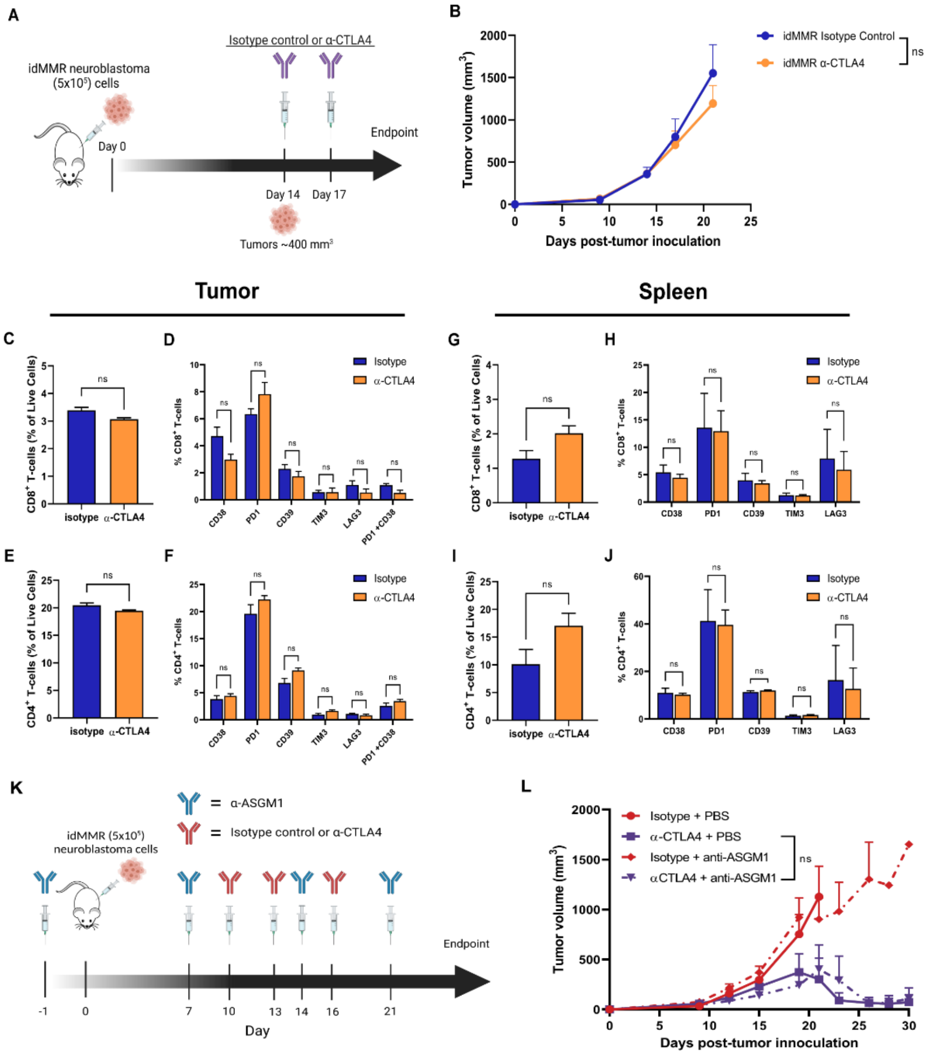

Figure S7: Anti-CTLA4 treatment does not induce inhibitory molecules on T-cells in a failure setting and the effect of anti-CTLA4 against idMMR neuroblastoma tumors is not NK cell dependent. On day 0, mice were injected subcutaneously with idMMR neuro-2a cells (500,000), then on days 14 and 17, they were either administered the isotype control or anti-CTLA4 antibody (n=3, n=5, respectively) (**A**). Tumor growth (**B**). On day 21, tumors and spleens were harvested and processed for analyzing TILs (left panel) and T-cells (right panel) using flow cytometry. Left panel, graphs are presented as a percentage of live cells, $CD3^{+}CD8^{+}$ TILs (**C**) or $CD3^{+}CD4^{+}$ TILs (**E**). Quantification of $CD38^{+}$, $PD1^{+}$, $CD39^{+}$, $TIM3^{+}$, $LAG3^{+}$, and $PD1^{+}CD38^{+}$ cells as a percentage of $CD8^{+}$ (**D**) and $CD4^{+}$ (**F**) T-cells from staining tumor samples. Data represent mean of technical replicates. Right panel, graphs are presented as a percentage of live cells, $CD3^{+}CD8^{+}$ T-cells (**G**) or $CD3^{+}CD4^{+}$ T-cells (**I**). Quantification of $CD38^{+}$, $PD1^{+}$, $CD39^{+}$, $TIM3^{+}$, $LAG3^{+}$ cells as a percentage of $CD8^{+}$ (**H**) and $CD4^{+}$ (**J**) T-cells from stained splenocyte samples. Experimental design and tumor growth of idMMR neuro-2a-bearing A/J mice treated with isotype control, anti-asialo GM1 (α-ASGM1) for NK cell depletion, anti-CTLA4, or anti-CTLA4 with α-ASGM1 (**K, L**). Statistical analyses were performed using the unpaired two-tailed Student's t-test or two-way ANOVA with Sidak's multiple comparisons test, ns (not significant), error bars indicate SEM.
