# Supplementary material for: Inducing mismatch repair deficiency sensitizes immune-cold neuroblastoma to anti-CTLA4 and generates broad anti-tumor immune memory
Source: Mol Ther. 2022 Sep 6;31(2):535–51. doi: 10.1016/j.ymthe.2022.08.025 (PMC9931548; doi:10.1016/j.ymthe.2022.08.025)
Supplement: Table S1. Key resources table [file mmc2.pdf]

Key Resources Table

REAGENT or RESOURCE	SOURCE	IDENTIFIER
Antibodies and dyes		
Brilliant Violet 711™ anti-mouse CD3 Antibody (clone: 17A2)	Biolegend	Cat# 100241
Alexa Fluor® 700 anti-mouse CD4 Antibody (clone: GK1.5)	Biolegend	Cat# 100536
PerCP/Cyanine5.5 anti-mouse CD8a Antibody (clone: 53-6.7)	Biolegend	Cat# 100734
PE/Dazzle™ 594 anti-mouse CD279 (PD-1) Antibody (clone: 29F.1A12)	Biolegend	Cat# 109116
PE/Cyanine7 anti-mouse CD223 (LAG-3) Antibody (clone: C9B7W)	Biolegend	Cat# 125226
PE anti-mouse CD366 (Tim-3) Antibody (clone: B8.2C12)	Biolegend	Cat# 119703
APC anti-mouse TIGIT (Vstm3) Antibody (clone: IG9)	Biolegend	Cat# 156106
Alexa Fluor® 647 anti-mouse CD39 Antibody (clone: Duha59)	Biolegend	Cat# 143808
Brilliant Violet 421™ anti-mouse CD107a (LAMP-1) Antibody (clone: 1D4B)	Biolegend	Cat# 121618
Alexa Fluor® 488 anti-mouse CD38 Antibody (clone: 90)	Biolegend	Cat# 102714
Brilliant Violet 605™ anti-mouse CD152 Antibody (clone: UC10-4B9)	Biolegend	Cat# 106323
Zombie NIR™ Fixable Viability Kit	Biolegend	Cat# 423106
PE/Cyanine7 anti-mouse CD3ε Antibody (clone: 145-2C11)	Biolegend	Cat# 100320
PerCP/Cyanine5.5 anti-mouse CD8a Antibody (clone: 53-6.7)	Biolegend	Cat# 100734
PE anti-mouse CD39 Antibody (clone: Duha59)	Biolegend	Cat# 143804
Zombie Green™ Fixable Viability Kit	Biolegend	Cat# 423111
anti-mouse CD16/32 antibody (Clone: 93)	Biolegend	Cat:101302
Anti-GT1b Ganglioside Antibody (clone: GT1b-2b)	MilliporeSigma	SKU# MAB5608
Rat IgGa Isotype control (anti-PD1 isotype control)	BioXCell	Cat# BP0089
Anti-PD1 antibody (clone: RMP1-14)	BioXCell	Cat# BP0146
Polyclonal Syrian Hamster IgG (anti-CTLA4 isotype control)	BioXCell	Cat# BP0087
Anti-CTLA4 antibody (clone: 9H10)	BioXCell	Cat# BP0131
Anti-CD4 antibody (clone: GK1.5)	BioXCell	Cat# BE0003-1
Chemicals, peptides, and recombinant proteins		

Biological samples		
TCGA Project: Primary tumor samples: Stomach adenocarcinoma		http://firebrowse.org/?cohort=STAD
TCGA Project: Primary tumor samples: Colorectal adenocarcinoma		http://firebrowse.org/?cohort=COADREAD
TARGET Project Primary tumor samples: Neuroblastoma		http://firebrowse.org/?cohort=COADREAD
Microarray neuroblatoma samples		GEO: GSE49710
Critical commercial assays		
Tumor Dissociation Kit, mouse	Miltenyi Biotec	130-096-730
Mouse IFN-β ELISA kit (serum, plasma, TCM)	PBL Assay Science	42410-2
RNeasy Mini kit	Qiagen	74104
Experimental models: Cell lines		
Neuro-2a cells	ATCC	CCL-131
Experimental models: Organisms/strains		
Mouse: A/J	The Jackson Laboratory	RRID:IMSR_JAX:000646
Mouse: SCID Hairless Outbred (SHO®) Mouse: Crl:SHO-PrkdcscidHrhr	Charles River	474
Oligonucleotides		
Guide RNA sc-421660 A – sense: TACCTCACCACGAAAGCCAT sc-421660 B – sense: TCACCGTGATCAGGGTGCCC sc-421660 C – sense: ACTTACGGTTGATGAAGAGT	Santa cruz biotechnology	sc-421660
Software and algorithms		
Python		https://www.python.org/
Pandas		https://pandas.pydata.org/
Lifelines		https://lifelines.readthedocs.io/en/latest/
Seaborn		https://seaborn.pydata.org/
GraphPad Prism		Version 9.3.1
FlowJo		Version 10.6.2
