# Supplementary material for: Inducing mismatch repair deficiency sensitizes immune-cold neuroblastoma to anti-CTLA4 and generates broad anti-tumor immune memory
Source: Mol Ther. 2022 Sep 6;31(2):535–51. doi: 10.1016/j.ymthe.2022.08.025 (PMC9931548; doi:10.1016/j.ymthe.2022.08.025)
Supplement: Document S2. Article plus supplemental information [file mmc3.pdf]

Molecular Therapy
Original Article

Inducing mismatch repair deficiency sensitizes immune-cold neuroblastoma to anti-CTLA4 and generates broad anti-tumor immune memory

Mikal El-Hajjar,[1,5,6] Lara Gerhardt,[2] Megan M Y Hong,[2] Mithunah Krishnamoorthy,[2,7] Rene Figueredo,[3] Xiufen Zheng,[1,2,3,4] James Koropatnick,[1,3,5] and Saman Maleki Vareki[2,3,5]

[1]Department of Microbiology and Immunology, Western University, London, ON, Canada; [2]Department of Pathology and Laboratory Medicine, Western University, London, ON, Canada; [3]Department of Oncology, Western University, London, ON, Canada; [4]Department of Surgery, Western University, London, ON, Canada; [5]London Regional Cancer Program, Lawson Health Research Institute, London, ON, Canada

Immune checkpoint blockade can induce potent and durable responses in patients with highly immunogenic mismatch repair-deficient tumors; however, these drugs are ineffective against immune-cold neuroblastoma tumors. To establish a role for a T cell-based therapy against neuroblastoma, we show that T cell and memory T cell-dependent gene expression are associated with improved survival in high-risk neuroblastoma patients. To stimulate anti-tumor immunity and reproduce this immune phenotype in neuroblastoma tumors, we used CRISPR-Cas9 to knockout *MLH1*—a crucial molecule in the DNA mismatch repair pathway—to induce mismatch repair deficiency in a poorly immunogenic murine neuroblastoma model. Induced mismatch repair deficiency increased the expression of proinflammatory genes and stimulated T cell infiltration into neuroblastoma tumors. In contrast to adult cancers with induced mismatch repair deficiency, neuroblastoma tumors remained unresponsive to anti-PD1 treatment. However, anti-CTLA4 therapy was highly effective against these tumors. Anti-CTLA4 therapy promoted immune memory and T cell epitope spreading in cured animals. Mechanistically, the effect of anti-CTLA4 therapy against neuroblastoma tumors with induced mismatch repair deficiency is CD4^{+} T cell dependent, as depletion of these cells abolished the effect. Therefore, a therapeutic strategy involving mismatch repair deficiency-based T cell infiltration of neuroblastoma tumors combined with anti-CTLA4 can serve as a novel T cell-based treatment strategy for neuroblastoma.

INTRODUCTION

Neuroblastoma is the most common extracranial cancer in children, with greater than 50% mortality in high-risk patients. High-risk neuroblastoma classification depends on a number of factors, including *MYCN* amplification status, disease stage, patient age at the time of diagnosis, chromosomal aberrations, tumor histology, and tumor cell ploidy.[1] *MYCN* amplification is present in approximately 20% of high-risk neuroblastoma cases, which is associated with suppressing the functional immunity pathways and immune cell infiltration into tumors.[2,3] Relapse in high-risk neuroblastoma patients poses a considerable therapeutic challenge and highlights the lack of long-term protective anti-tumor T cell-based response in these patients. Currently, anti-ganglioside 2 (GD2) drugs remain the only approved immunotherapy for neuroblastoma. However, response to such drugs in high-risk patients diminishes over time.[4] T cell-based immunotherapies may provide a suitable alternative, as T cells exhibit memory responses crucial for sustained remission in advanced cancers.[5] However, these therapies have had limited success in neuroblastoma due to low neoepitope expression and sparse T cell infiltration into neuroblastoma tumors.[6] For example, neuroblastoma patients treated with CAR T cells targeting GD2 have shown no objective clinical response to treatment.[7] Furthermore, immune checkpoint blockade (ICB), which leverages pre-existing anti-tumor T cells, is not currently used to treat most childhood malignancies, including neuroblastoma, because of limited success.[8]

In contrast to immune-cold neuroblastoma, tumors with mutations in DNA mismatch repair (MMR) genes often show increased immunogenicity, higher T cell infiltration, and improved response to ICBs.[9,10] Recently, inducing MMR deficiency has been proposed as a therapeutic strategy to sensitize adult solid tumors to immunotherapy, regardless of the tumor's tissue of origin or mutation background.[11] Therefore, therapeutic strategies, including small-molecule inhibitors, are being developed to induce MMR deficiency in adult solid tumors.[12,13] However, such therapeutic strategies are not yet pursued in childhood cancers, particularly highly immune-cold cancers such as neuroblastoma, where an MMR-deficient (dMMR) phenotype is not common.[14] Therefore, inducing MMR deficiency

Received 2 May 2022; accepted 30 August 2022;
https://doi.org/10.1016/j.ymthe.2022.08.025.

[6]Present address: Department of Medicine, Faculty of Medicine and Health Sciences, McGill University Health Centre; Montreal, QC, Canada
[7]Present address: Pfizer Inc., Mississauga, ON, Canada
Correspondence: Saman Maleki Vareki, Department of Pathology and Laboratory Medicine, Western University, London, ON, Canada.
E-mail: saman.malekivareki@lhsc.on.ca

This is an open access article under the CC BY license (http://creativecommons.org/licenses/by/4.0/).

as a therapeutic strategy in this immune-cold and low tumor mutational burden (TMB) cancer could transform the immune phenotype of neuroblastoma tumors and sensitize them to T cell-based immunotherapies. However, it is currently unknown whether this therapeutic approach could render neuroblastoma—a highly immunotherapy-refractory tumor—sensitive to ICBs in a manner similar to other adult malignancies.

Here we show that high expression of T cell and memory T cell-related genes in high-risk neuroblastoma patients is associated with improved survival, which provides a rationale for developing T cell-based interventions in this cancer. Furthermore, we reproduced this phenotype by inducing MMR deficiency in a poorly immunogenic syngeneic murine neuroblastoma tumor model. Our *in vivo* studies demonstrate increased anti-tumor T cell responses against neuroblastoma tumors with induced MMR deficiency compared with wild-type mismatch repair-proficient (pMMR) neuroblastoma. Notably, we show that, despite the higher immunogenicity of induced dMMR (idMMR) neuroblastoma tumors, anti-programmed cell death 1 (PD1) therapy does not elicit a therapeutic effect against such tumors similar to adult malignancies treated with this treatment strategy.[11] This can be partly due to T cell exhaustion and dysfunction resulting from lower IFN signaling and the downregulation of beta-2-microglobulin (B2M) and MHC class I in these tumors.

In contrast, we found that anti-cytotoxic T lymphocyte-associated protein-4 (CTLA4) therapy increased tumor-specific T cells and was highly effective against idMMR neuroblastoma tumors. Importantly, anti-CTLA4 treatment induced an immunological memory response with epitope spreading in cured mice, resulting in complete rejection of tumors following rechallenge with immunogenic idMMR or poorly immunogenic pMMR neuroblastoma tumors. Mechanistically, the effect of anti-CTLA4 therapy is dependent on $CD4^+$ T cells, as depletion of these T cells abolishes the protective effect of anti-CTLA4 therapy. These studies establish a new treatment strategy that eradicates immune-cold neuroblastoma tumors and generates a long-term and broad immune memory response by inducing MMR deficiency in neuroblastoma tumors in combination with an ICB treatment.

RESULTS

Increased T cell-related gene transcript levels are associated with improved survival in high-risk neuroblastoma patients

Neuroblastoma is an immune-cold cancer with recurrence commonly seen in high-risk patients. We aimed to elucidate the clinical importance of T cell markers in neuroblastoma to assess the need for a T cell-based treatment strategy in this cancer. Therefore, we mined the "Therapeutically Applicable Research to Generate Effective Treatments" (TARGET) database containing RNA-seq data from neuroblastoma tumor samples and relevant clinical information, including patient survival. First, we compared the overall survival of low-risk versus high-risk neuroblastoma patients. As expected, high-risk neuroblastoma patients had significantly worse survival than low-risk patients (Figure S1A). We then analyzed the prognostic role of several immune markers in high-risk neuroblastoma patients (Figures 1 and S1B–S1N) and found a prognostic role for the CD8 T cell marker and genes associated with critical T cell functions in these patients (Figure 1A). IL-2 has a well-described role in the expansion, effector function, and memory responses of $CD4^+$ and $CD8^+$ T cells.[15] *IL2* gene expression was also positively correlated with improved survival in these patients (Figure 1B).

Similarly, IL-6 plays a role in T cell differentiation and cytokine production and was associated with a favorable outcome in high-risk neuroblastoma (Figure 1C). Following re-exposure to the same antigen, memory T cells exhibit increased cytokine secretion, proliferation, and cytotoxicity. These cells are crucial for long-term disease control and durable immunity that is lacking in relapsed neuroblastoma patients.[16] We found that memory T cell-associated genes, *IL7R*, *ID2*, *ID3*, and *CXCR3* were associated with improved clinical outcomes in these patients (Figures 1D–1G). Given the improved survival of low-risk neuroblastoma patients compared with high-risk patients (Figure S1A), we also explored whether the above immune markers that had a prognostic role in high-risk patients were also expressed in low-risk neuroblastoma patients. With the exception of *ID2*, all the other immune genes (*CD8a*, *IL2*, *IL7R*, *IL6*, *ID3*, and *CXCR3*) were also highly expressed in low-risk neuroblastoma patients further confirming the significance of these immune genes in neuroblastoma cases with favorable outcomes (Figures S2A–S2G). We then focused on a subset of high-risk neuroblastoma patients with *MYCN* amplification to determine whether the *MYCN* amplification status in patients uniquely drives the improved survival associated with T cell gene expression. Therefore, we examined overall survival based on T cell-associated gene expression in patients with and without *MYCN* amplification. We did not find any *MYCN* amplification-driven effect on the prognostic role of those immune markers, which further highlights the independent significance of T cell-related gene expression on neuroblastoma patient outcomes (Figures S3A–S3N).

MLH1 downregulation is common among MMR-deficient and microsatellite instability-high cancers that exhibit higher T cell infiltration

Microsatellite instability-high (MSI-h) and mismatch repair-deficient (dMMR) cancers often show high T cell infiltration (Figures S4A and S4B) and improved response to ICBs.[17] Therefore, inducing MMR deficiency in tumor cells has been proposed as a potential therapeutic strategy.[11] Therefore, we decided to study whether inducing MMR deficiency in immune-cold neuroblastoma could increase its immunogenicity. MMR deficiency is more common among colorectal cancer patients, while patients with functional DNA repair machinery are typically classified as microsatellite stable (MSS).[18] To determine which component of the MMR pathway should be targeted in neuroblastoma tumors, we mined The Cancer Genome Atlas (TCGA) database to investigate the expression of MMR genes in MSI-h colorectal and stomach adenocarcinoma patients. We found that *MLH1* gene expression was significantly lower in MSI-h samples than in MSS and normal samples (Figure S4C). Other MMR genes, such as

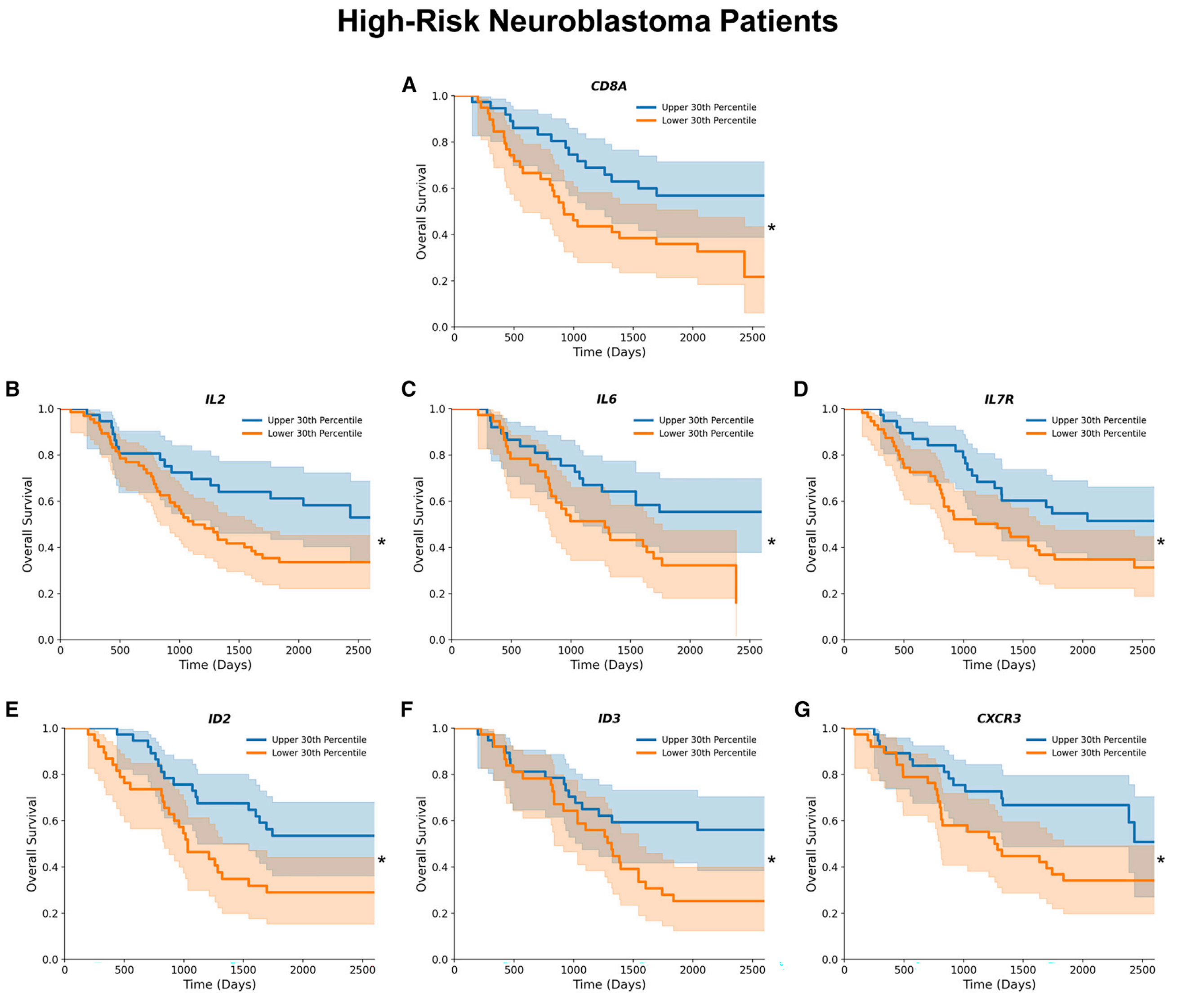

Figure 1. T cell-related gene expression correlates with improved survival in high-risk neuroblastoma patient

(A–G) Kaplan-Meier survival analysis by log rank test of high-risk neuroblastoma patients stratified according to upper and lower 30th percentile gene expression of (A) CD8A (n = 38, n = 38), (B) IL-2 (n = 38, n = 66), (C) IL-6 (n = 38, n = 38), (D) IL-7R (n = 38, n = 56, (E) ID2 (n = 38, n = 38), (F) ID3 (n = 38, n = 38), (G) CXCR3 (n = 38, n = 38). *$p \leq 0.05$.

MSH2 and *MSH6*, did not have significantly different expression levels among MSI-h and MSS samples (Figures S4D and S4E). Whereas another MMR gene, *PMS2*, had lower gene expression in MSI-h samples (Figure S4F). To further explore which MMR genes are associated with a high TMB MSI-h phenotype, we repeated this analysis in a second dataset (STAD) and obtained similar results. However, in this dataset, *PMS2* gene expression was not significantly different between MSS and MSI-h samples, whereas *MLH1* gene expression remained significantly downregulated in MSI-h samples (Figures S4G–S4J). These findings highlight the importance of the *MLH1* gene in conferring the MSI-h phenotype and potentially the immunogenicity of these tumors. Therefore, we targeted the *MLH1* gene to induce MMR deficiency in neuroblastoma tumors.

To induce a dMMR phenotype in neuroblastoma tumors, we knocked out the *MLH1* gene in a poorly immunogenic syngeneic murine neuroblastoma cell line, neuro-2a, using the clustered regularly interspaced short palindromic repeats-associated protein (CRISPR-Cas9) technology. We selected multiple clones and compared *MLH1* gene and protein expression levels in the knockout clones (C4, C5, C10, C12) to the parental cells (wild-type neuro-2a cells) (Figures S4K and S4L). To ensure that both the MMR-proficient (pMMR) and induced dMMR (idMMR) neuroblastoma cells had similar baseline growth kinetics, we conducted an Incucyte cell proliferation assay with multiple clones and found no significant differences in cell growth rates between the idMMR and the WT parental pMMR neuroblastoma cells (Figure S4M). We then chose a clone with an

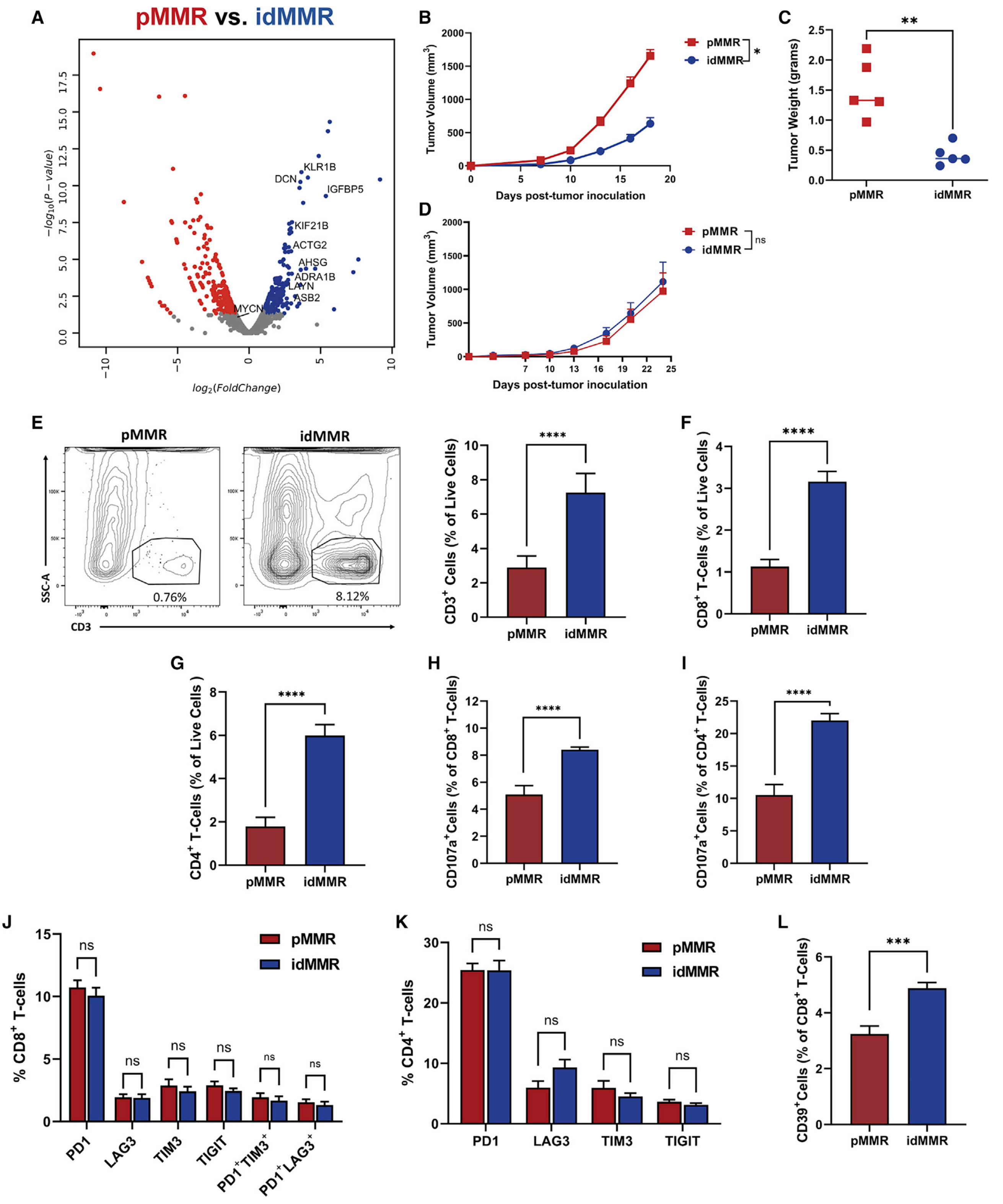

(legend on next page)

unsuccessful CRISPR-Cas9 knockout of the *MLH1* gene with similar growth rates to clones with successful *MLH1* knockout as our pMMR control for comparison purposes. The pMMR (C12) and idMMR (C4) neuroblastoma cell lines have similar baseline growth kinetics *in vitro* (Figure S4N) and are suitable for studying immune-based anti-tumor responses in a syngeneic mouse model.

Inducing MMR deficiency in neuroblastoma tumors stimulates immune-based anti-tumor responses

To study the effect of inducing MMR deficiency on the immunogenicity of neuroblastoma tumors, we compared the transcriptomic profile of idMMR and pMMR neuroblastoma cells after 8 or 14 weeks in culture. We found increased expression levels of multiple genes associated with higher immunogenicity in idMMR cells, including genes negatively correlated with *MLH1* expression and positively correlated with higher TMB and immune score in solid tumors (*KLRB1B*),[19] (*ADRA1B*),[20] inactivation of TGF-β (*DCN*),[21] (*AHSG*),[22] preferential $CD4^+$ T cell infiltration of tumors (*KIF21B*),[23] *IGFBP5*,[24] and immunological activation and immune cell infiltration in tumors (*ACTG2*),[25] (*LAYN*),[26] (*ASB2*)[27] (Figure 2A). These transcriptomic changes suggest heightened immunogenicity after inducing MMR deficiency in idMMR neuroblastoma cells. We also compared the changes in *MYCN* expression between idMMR and pMMR cells and found no changes between the cell lines (Figure 2A).

To validate our RNA-seq findings in an *in vivo* model, we compared tumor growth of pMMR and idMMR cells in immunocompetent A/J mice. idMMR neuroblastoma tumors exhibited significantly reduced growth compared with pMMR tumors (Figure 2B). Furthermore, tumor weights recorded at the experimental endpoint (day 18 post tumor injection) also confirmed the reduction of tumor burden in idMMR neuroblastoma tumors (Figure 2C). Notably, the effect of inducing MMR deficiency on tumor regression was immune-mediated as idMMR and pMMR neuroblastoma tumors grew at the same rate in immunodeficient SHO mice, which lack a functional adaptive immune system (Figure 2D). We also compared the growth of idMMR tumors in male and female A/J mice and found no difference in tumor growth rate between male and female animals (Figure S4O). Therefore, we focused the rest of our *in vivo* experiments on female animals.

To characterize the immune mechanisms underlying the tumor growth inhibition of idMMR neuroblastoma tumors, we isolated and analyzed tumor-infiltrating lymphocytes (TILs). Neuroblastoma tumors with induced MMR deficiency had an increase in overall T cell infiltration (Figure 2E), mainly $CD8^+$ and $CD4^+$ T cells (Figures 2F, 2G, and S5E), with increased cytotoxicity characterized by CD107a expression (Figures 2H and 2I).[28] Furthermore, T cells in idMMR tumors did not exhibit increased expression of single or multiple inhibitory receptors, including PD1, LAG3, TIM3, and TIGIT, which indicates that inducing MMR deficiency in neuroblastoma tumors and the potential exposure to more antigens does not induce exhaustion in these T cells (Figures 2J, 2K, and S5F). We also observed more $CD39^+$ tumor-specific T cells among $CD8^+$ TILs isolated from idMMR tumors (Figure 2L).[29,30]

Anti-PD1 is not effective against idMMR neuroblastoma tumors with reduced MHC class I levels

To determine whether inducing MMR deficiency in neuroblastoma sensitizes such tumors to anti-PD1, as shown in other adult malignancies, we treated both pMMR and idMMR neuroblastoma tumor-bearing mice with anti-PD1 when tumors became palpable (~day 10) and assessed the toxicity of the treatment and found no significant changes in mouse body weight (Figures 3A and S5A). As expected from an immunologically cold cancer, pMMR neuroblastoma tumors were refractory to anti-PD1 therapy (Figure 3B). Surprisingly, anti-PD1 treatment did not confer any additional therapeutic benefit beyond the reduced tumor growth resulting from induced MMR deficiency in neuroblastoma tumors (Figures 3C and 3D).

Neuro-2a cells do not express PD1, nor does the induction of MMR deficiency cause aberrant expression of PD1 (Figure S4P). Therefore, to better understand why anti-PD1 treatment was ineffective against idMMR neuroblastoma tumors, we decided to examine the immune features of idMMR neuroblastoma tumors that can impact their response to anti-PD1 treatment.

Neuroblastoma is known to have low expression of MHC class I,[31] which can contribute to its insensitivity to T cell-based therapies. Moreover, mutations in MHC class I genes are common in immune-inflamed tumors with increased lymphocyte infiltration, such as in MSI-h colorectal cancer.[32,33] Therefore, we studied whether inducing MMR deficiency in neuroblastoma tumors recapitulates some of the negative tumor-intrinsic features of MSI-high cancers, such as lower MHC class I levels, which can potentially play a role in the lack of therapeutic efficacy of anti-PD1 treatment against idMMR neuroblastoma tumors. We, therefore, analyzed MHC class

Figure 2. Inducing MMR deficiency in neuroblastoma tumors stimulates a robust anti-tumor immune response

(A–L) pMMR and idMMR neuroblastoma cells were cultured for 8 weeks *in vitro* and analyzed using bulk mRNA sequencing. Data are represented as a volcano plot illustrating differential gene expression of pMMR versus idMMR neuroblastoma cells (A). Genes significantly upregulated (log fold change > 1) and downregulated (log fold change < −1) in the pMMR and idMMR groups are represented as red and blue, respectively. pMMR and idMMR neuroblastoma cells (5×10^5) were inoculated subcutaneously into A/J mice, and tumor growth (B) (n = 15, n = 17) and weight (C) (n = 5, n = 5) were measured. pMMR and idMMR neuroblastoma cells (5×10^5) were inoculated subcutaneously into SHO immunodeficient mice and tumor growth was monitored over a 24-day period (D). A/J mice were sacrificed at day 18, and tumors were pooled together for each group. Single-cell suspension from sampled were generated and stained for flow cytometry analysis. Cells are presented as a percentage of live cells. Results are representative of 3 independent experiments n = 15, n = 17. Quantification of $CD3^+$ (E), $CD8^+$ (F), $CD4^+$ cells (G), and $CD107a^+$ (H and I), $PD1^+$, $LAG3^+$, $TIM3^+$, $TIGIT^+$, $TIM3^+PD1^+$, $LAG3^+PD1^+$ $CD8^+$, and $CD4^+$ T cells (J and K). Quantification of $CD39^+$ $CD8^+$ T cells in tumors (L). See also Figure S5. Statistical analysis was performed using the unpaired two-tailed Student's t test. *p $\leq$ 0.05, **p $\leq$ 0.01, ***p $\leq$ 0.001, ****p $\leq$ 0.0001; ns (not significant).

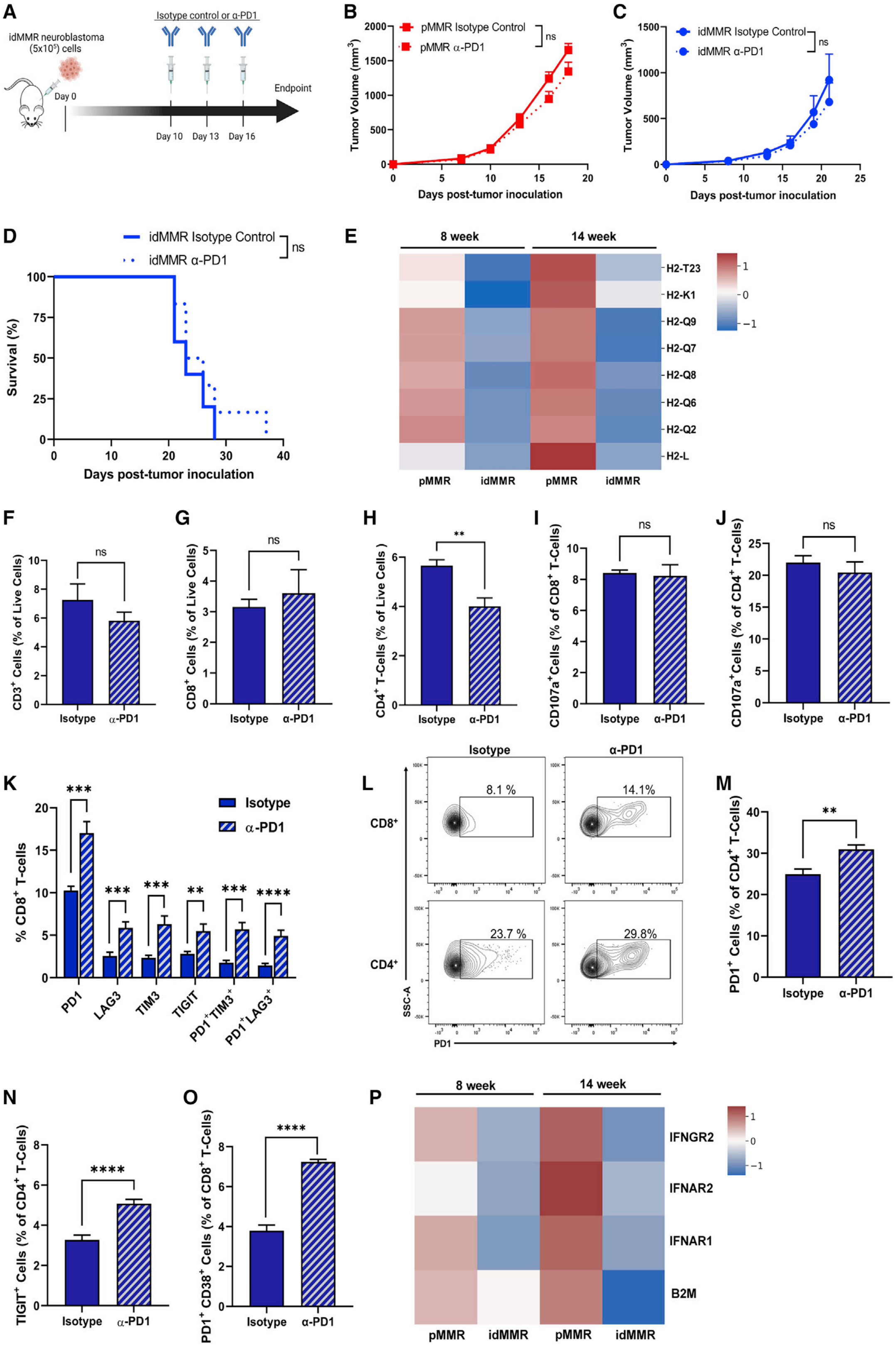

(legend on next page)

I-related gene signatures of pMMR and idMMR cells. Intriguingly, we found that MHC class I genes were downregulated in idMMR cells both at 8 and 14 weeks of culture compared with pMMR neuroblastoma cells (Figure 3E).

Anti-PD1 therapy induces T cell exhaustion and dysfunction in idMMR neuroblastoma tumors

It has been reported that lung cancer patients with acquired resistance to anti-PD1 therapy have a low MHC class I surface expression and an inflammatory tumor microenvironment, marked by increased expression of immune checkpoint molecules on T cells.[34] idMMR neuroblastoma cells exhibited a decrease in MHC class I gene expression, and anti-PD1 therapy did not confer any additional therapeutic benefit, despite increased $CD8^+$ T cell infiltration in response to MMR induction in these tumors (Figure 2F). Therefore, we further analyzed the TILs isolated from idMMR tumors after anti-PD1 treatment to examine whether T cells show increased expression of immune checkpoint molecules similar to T cells isolated from lung tumors with low MHC class I levels in patients.[34]

Anti-PD1 therapy in idMMR tumor-bearing mice did not increase overall T cell infiltration or $CD8^+$ T cell infiltration into the tumors (Figures 3F and 3G). We also observed reduced $CD4^+$ T cell levels in idMMR neuroblastoma tumors treated with anti-PD1 (Figure 3H). Furthermore, anti-PD1 treatment did not increase effector $CD107a^+CD8^+$ or $CD107a^+CD4^+$ T cells which are crucial for effective tumor cell killing in the tumor microenvironment (Figures 3I and 3J). Notably, we observed an increase of $CD8^+$ T cells from idMMR tumors expressing inhibitory receptors: PD1, LAG3, TIM3, and TIGIT (Figures 3K and 3L), and PD1 and TIGIT on $CD4^+$ T cells after anti-PD1 therapy, suggesting an induction of an exhausted phenotype (Figures 3L–3N). We also observed a higher proportion of terminally exhausted and dysfunctional $CD8^+$ T cells ($PD1^+CD38^+$) after anti-PD1 treatment in idMMR neuroblastoma tumors (Figure 3O).

IFN signaling gene expression is downregulated in neuroblastoma tumors with induced MMR deficiency

Our data show that induced MMR deficiency in neuroblastoma tumors increases the expression of several proinflammatory genes in these tumors and stimulates T cell-mediated anti-tumor immunity that delays tumor growth. However, we also observed lower MHC class I-related gene expression in idMMR neuroblastoma cells that could negatively affect their response to anti-PD1 treatment.

MHC class I genes are among IFN signaling genes, and IFNs regulate their expression in cells.[35,36] Therefore, we broadly analyzed the expression of type I and type II IFN genes from pMMR and idMMR tumor cells cultured *in vitro* for 8 and 14 weeks. We found a trend of decreased IFN-related gene expression in idMMR cells (Figures S6A and S6B). We then, more specifically, studied the effect of inducing MMR deficiency on the expression of IFN receptors, which are critical for IFN signaling. We show that idMMR cells have decreased expression of critical interferon receptors, such as *IFNGR2*, *IFNR1*, and *IFNR2* (Figure 3P). Moreover, pathway enrichment analysis of the transcriptome of pMMR and idMMR cells showed a downregulation of the JAK-STAT signaling pathway in idMMR cells, further supporting lower IFN signaling in these cells (Figure S6C). Although most tumors have lower IFN signaling, it has been previously reported that MSI-h lung and colorectal tumors can produce IFN-β that increases their immunogenicity.[9] To directly determine whether lower IFN signaling and downregulated JAK-STAT pathway in idMMR neuroblastoma affects their ability to produce IFN-β, we quantified IFN-β levels in the supernatant of cultured idMMR neuroblastoma cells by ELISA. IFN-β was not detected in the culture medium (Figure S6D), further experimentally confirming the lower IFN signaling in idMMR neuroblastoma tumors that can negatively impact their MHC class I expression and potential response to anti-PD1 therapy.

Neuroblastoma tumors with induced MMR deficiency exhibit lower B2M gene expression but respond to anti-CTLA4 therapy

MSI-h colorectal tumors commonly show biallelic disruption of the *B2M* gene that affects their MHC class I surface expression and the effectiveness of anti-PD1 therapy.[37,38] Therefore, we compared *B2M* gene expression among pMMR and idMMR neuroblastoma cells. We found that *B2M* gene expression was also lower in idMMR neuroblastoma cells, which may further explain the lack of effectiveness of anti-PD1 therapy against these tumors, similar to clinical findings with MSI-h tumors (Figure 3P).[34]

It has been shown that MSI-h tumors with *B2M* gene deletion remain sensitive to ICB treatment in a $CD4^+$ T cell-dependent manner.[38] In our studies, we observed an increase in $CD4^+$ T cells in idMMR neuroblastoma tumors (Figure 2G); therefore, we decided to treat the

Figure 3. Anti-PD1 therapy triggers T cell exhaustion and fails to enhance anti-tumor response against neuroblastoma tumors with induced MMR deficiency
(A–P) On day 0, mice were inoculated subcutaneously with 5×10^5 pMMR or idMMR neuroblastoma cells (n = 6, n = 7, respectively). Mice received isotype control or anti-PD1 (250 μg) intraperitoneally on day 10, 13, and 16, and tumors were harvested on day 18 (A). Tumor growth for pMMR and idMMR tumor-bearing mice (B and C) and survival for idMMR tumor-bearing mice was measured (D). Bulk mRNA sequencing data from pMMR and idMMR neuroblastoma cells cultured for 8 weeks (n = 1, n = 1) and 14 weeks (n = 1, n = 1) *in vitro* are represented as a heatmap displaying the relative gene expression (*Z* score by row) of MHC class I-related genes (E). idMMR tumors from each group were pooled before staining for flow cytometry to analyze TILs. Data are presented as a percentage of live cells, $CD3^+$ TILs (F), $CD3^+CD8^+$ TILs (G), or $CD3^+CD4^+$ TILs (H). Quantification of $CD107a^+$ cells as a percentage of $CD8^+$ (I) and $CD4^+$ (J) TILs. Quantification of $PD1^+$, $LAG3^+$, $TIM3^+$, $TIGIT^+$, $PD1^+TIM3^+$, $PD1^+LAG3^+$ cells as a percentage of $CD8^+$ TILs (K and L). Quantification of $PD1^+$ (L and M) and $TIGIT^+$ (N) cells as a percentage of $CD4^+$ TILs. Quantification of $PD1^+CD38^+$ $CD8^+$ TILs (O). Results are representative of 4 independent experiments (n = 25, n = 25). Bulk mRNA sequencing data from pMMR and idMMR neuroblastoma cells (n = 1, n = 1) cultured for 8 weeks (n = 1, n = 1) and 14 weeks (n = 1, n = 1) *in vitro* are represented as a heatmap displaying the relative gene expression (*Z* score by row) of IFN-related genes and *B2M* gene expression (P). See also Figure S6. Statistical analysis was performed using the unpaired two-tailed Student's t test. $^*p \leq 0.05$, $^{**}p \leq 0.01$, $^{***}p \leq 0.001$, $^{****}p \leq 0.0001$; ns, not significant, error bars indicate SEM.

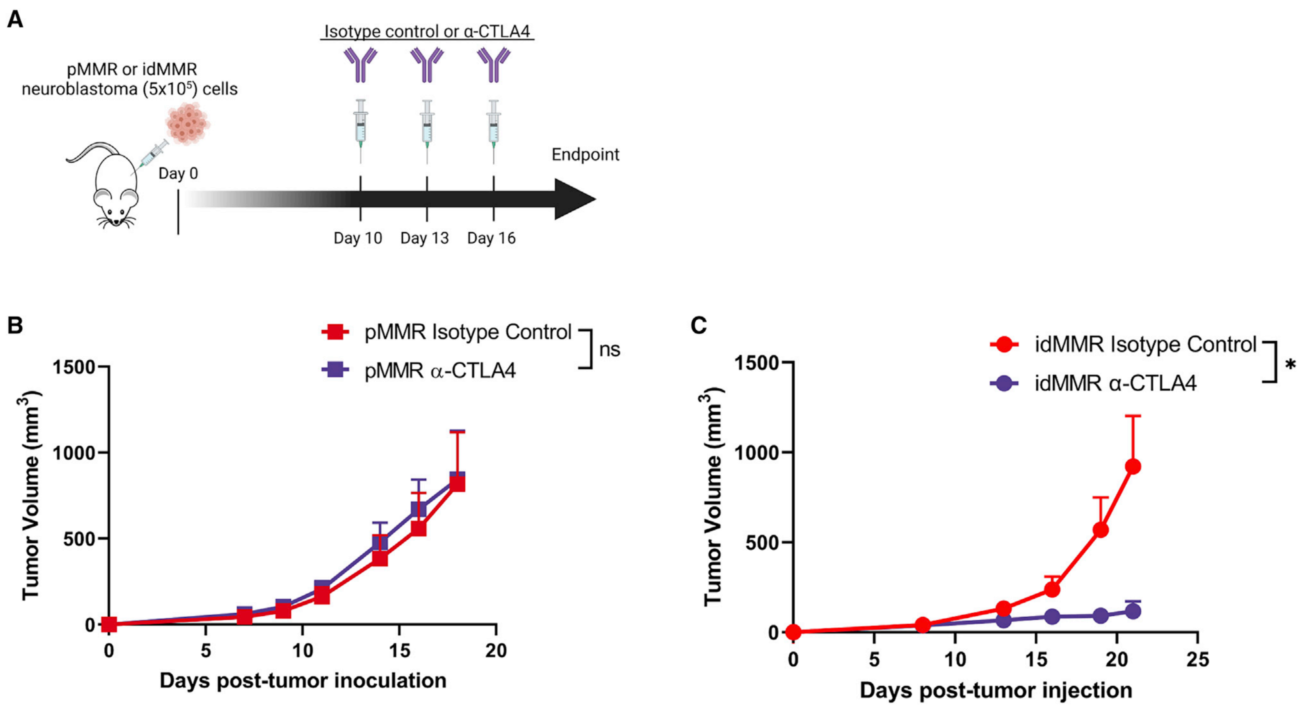

Figure 4. Induced MMR deficiency sensitizes neuroblastoma tumors to anti-CTLA4 therapy

(A–C) pMMR or idMMR cells neuroblastoma cells (5×10^5) were inoculated into immunocompetent A/J mice for the following experiments. Mice were treated with either isotype control or anti-CTLA (200 μg) at days 10, 13, 16, and sacrificed at ~ day 20 (A). Tumor growth of pMMR neuroblastoma tumor-bearing mice administered with isotype control or anti-CTLA4 (n = 4, n = 5, respectively) (B). Tumor growth of idMMR tumor-bearing mice given isotype control or anti-CTLA4 (n = 5, n = 7) (C). Statistical analyses were performed using the unpaired two-tailed Student's t test. *$p \leq 0.05$; ns (not significant), error bars indicate SEM.

tumor-bearing animals with an anti-CTLA4, which mainly depends on $CD4^+$ T cells for its effectiveness.[39,40] To this end, we treated pMMR and idMMR neuroblastoma tumor-bearing mice with anti-CTLA4 therapy to assess T cell-based anti-tumor immune responses (Figure 4A). This treatment effectively inhibited the growth of idMMR neuroblastoma tumors, and it cured ~70% of idMMR neuroblastoma tumor-bearing mice without inducing any significant toxicity in treated animals (Figures 4B, 4C, and S5B). Our findings demonstrate that induction of MMR deficiency in neuroblastoma tumors can sensitize them to anti-CTLA4 treatment, which primarily relies on $CD4^+$ T cells, but not anti-PD1 treatment, which mainly depends on $CD8^+$ T cells.[41]

Anti-PD1 and anti-CTLA4 combination therapy does not exhibit greater therapeutic efficacy than anti-CTLA4 monotherapy against idMMR neuroblastoma tumors

Our data showed that, unlike other adult cancer murine models such as melanoma or colorectal cancer,[11] anti-PD1 therapy was ineffective against neuroblastoma tumors with induced MMR deficiency. However, since these tumors showed sensitivity to anti-CTLA4 therapy, we decided to combine anti-PD1 and anti-CTLA4 treatment to examine whether the combination strategy can offer a greater therapeutic effect.

Therefore, we inoculated the 8-week pMMR or idMMR neuroblastoma cells into A/J immunocompetent mice and treated them with anti-PD1, anti-CTLA4, or a combination of both antibodies (Figure 5A). Anti-PD1 did not show any therapeutic effect (Figure 5B), similar to our previous findings (Figure 5B). While anti-CTLA4 therapy controlled tumor growth effectively and cured ~70% of the animals, the combination therapy was not toxic in the animals but did not offer any further therapeutic benefit (Figures 5B and S5C).

It has been proposed that maintaining idMMR tumor cells in culture for an extended period (20–22 weeks) may sensitize tumors to anti-PD1 treatment by increasing their immunogenicity as cells accumulate more frameshift mutations.[11,42] Therefore, we decided to maintain idMMR neuroblastoma cells in culture for a longer yet therapeutically relevant time frame (14 weeks). We then assessed whether increased immunogenicity of idMMR neuroblastoma cells could overcome the lack of sensitivity to anti-PD1 therapy against idMMR neuroblastoma tumors. In addition, before growing these cells *in vivo* as tumors, we tested their proliferation rates *in vitro* and observed similar proliferation rates between idMMR neuroblastoma cells cultured for 8 and 14 weeks (Figure S6E). To validate the increased immunogenicity of idMMR cells with a more extended culture period (14 weeks), we inoculated 8- or 14-week idMMR neuroblastoma cells into immunocompetent mice and monitored animal survival. Mice inoculated with 14-week idMMR neuroblastoma cells had higher survival, indicating heightened immunogenicity of tumors

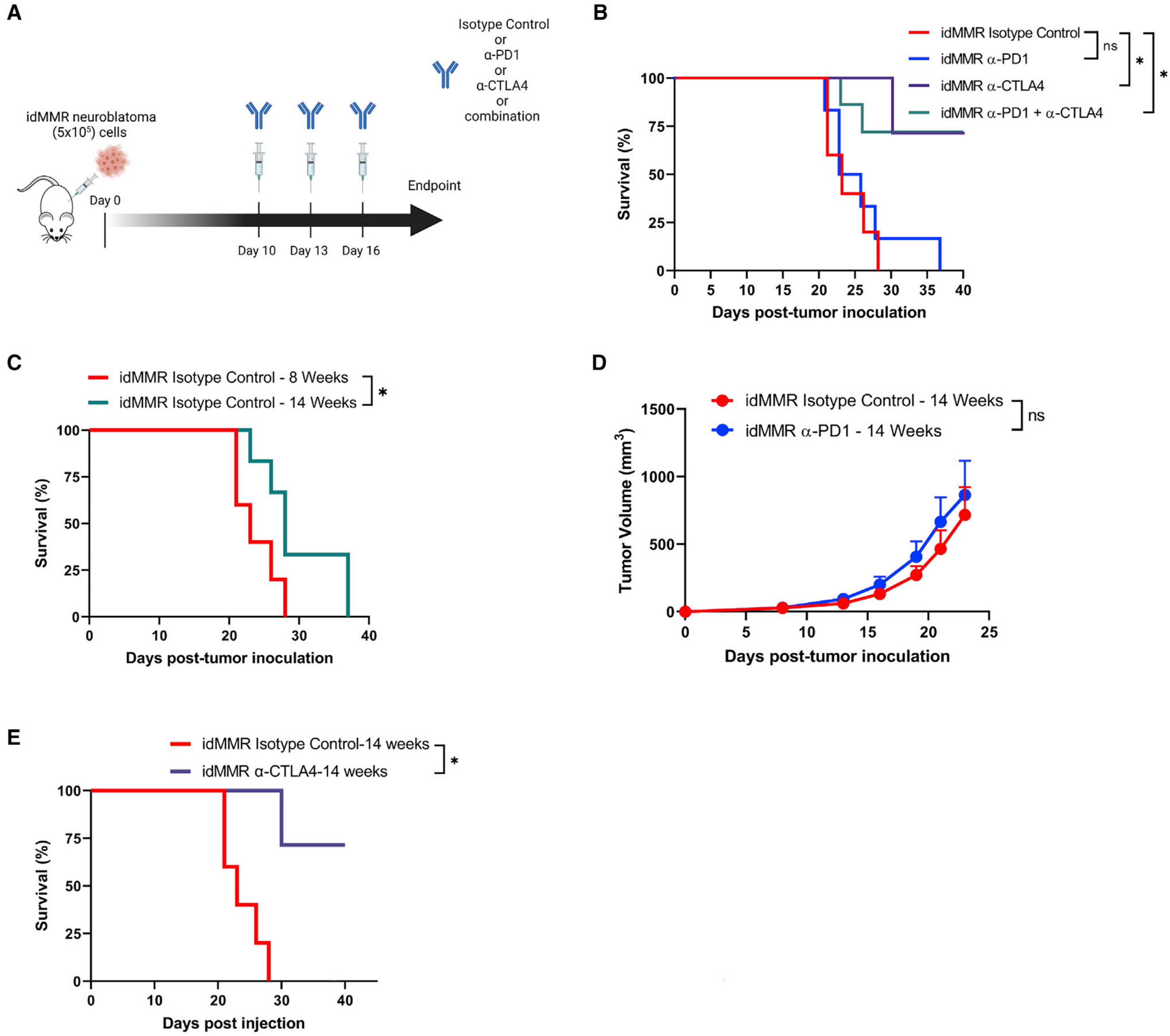

Figure 5. Anti-PD1 and anti-CTLA4 combination therapy does not provide additional therapeutic benefit against idMMR tumors

(A–E) idMMR neuroblastoma cells (5×10^5) grown for 8 weeks were then inoculated into immunocompetent A/J mice and treated with either isotype control, anti-PD1 (250 μg), or anti-CTLA (200 μg), or a combination of both, at days 10, 13, and 16, and sacrificed at around day 20 (A). A/J mice were inoculated with (5×10^5) idMMR neuroblastoma cells that were cultured for 8 weeks and treated with isotype control, anti-PD1, anti-CTLA4, or a combination of both anti-PD1 and anti-CTLA-4. Survival was assessed and displayed as Kaplan-Meier curves (n = 5, n = 6, n = 7, n = 7, respectively) (B). Mice were inoculated subcutaneously with idMMR neuroblastoma cells (5×10^5) grown for 8 or 14 weeks, respectively (n = 5, n = 6) and survival data are displayed using a Kaplan-Meier curve (C). idMMR neuroblastoma cells (5×10^5) grown for 14 weeks were then inoculated into immunocompetent A/J mice for the following experiments. Mice were treated with either isotype control, anti-PD1 (250 μg) or anti-CTLA (200 μg) at days 10, 13, and 16, and sacrificed at around day 20. Tumor growth of idMMR neuro-2a tumor-bearing mice administered with isotype control or anti-PD1 (n = 6, n = 7, respectively) was measured (D). Kaplan-Meier curves assessing survival of idMMR tumor-bearing mice treated with isotype control antibody or with anti-CTLA4 (n = 6, n = 7, respectively) (E). Statistical analyses were performed using the unpaired two-tailed Student's t test and log rank Cox test for survival. *$p \leq 0.05$; ns (not significant), error bars indicate SEM.

cultured for a prolonged period *in vitro* (Figure 5C). To study whether those tumors would be responsive to anti-PD1 therapy, idMMR 14-week neuroblastoma cells were inoculated in immunocompetent mice and treated with either isotype control or anti-PD1 therapy. Notably, these tumors remained resistant to anti-PD1 therapy (Figure 5D); however, they remained sensitive to anti-CTLA4 treatment (Figure 5E). These data indicate that idMMR neuroblastoma tumors remain unresponsive to anti-PD1 therapy, regardless of their state of

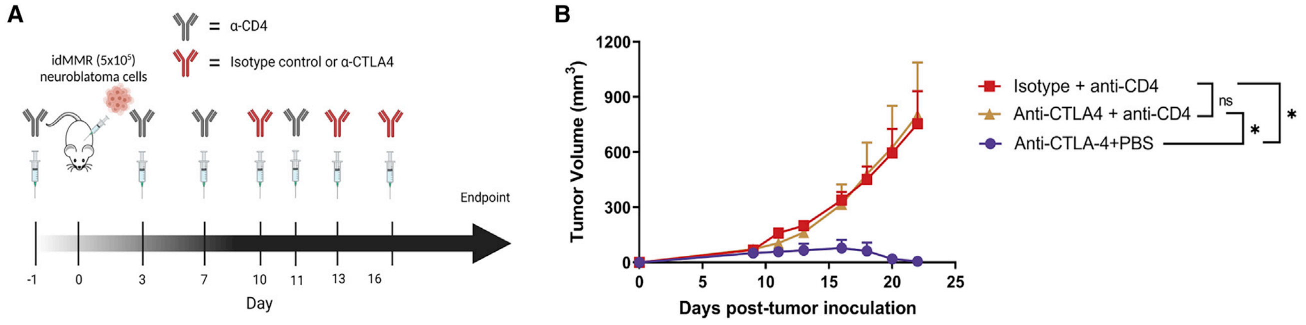

Figure 6. Therapeutic effect of anti-CTLA4 against idMMR tumors is dependent on CD4$^+$ T cells

(A and B) Immunocompetent A/J mice were treated with anti-CD4 (100 μg) or 100 μL PBS 24 h before tumor cell inoculation and on days 3, 7, and 11 after inoculation. Mice were inoculated with 8-week idMMR neuroblastoma cells (5×10^5) and treated with anti-CTLA4 (200 μg) on days 10, 13, and 16 (A). Tumors were measured from mice treated with isotype with anti-CD4, anti-CTLA-4 with PBS, and anti-CTLA4 with anti-CD4 (n = 6, n = 4, n = 7, respectively) (B). Statistical analysis was performed using unpaired two-tailed Student's t test. *$p \leq 0.05$, ns (not significant), error bars indicate SEM.

immunogenicity. However, such tumors can be eradicated by anti-CTLA4 therapy, with over 70% of mice cured after treatment.

Anti-CTLA4 treatment does not induce the expression of inhibitory molecules on T cells

Since the lack of therapeutic benefit of anti-PD1 treatment against idMMR neuroblastoma tumors was not associated with tumor immunogenicity but rather the induction of inhibitory checkpoint molecules on TILs, we decided to determine whether such a phenomenon was unique to anti-PD1 treatment or could be reproduced with anti-CTLA4 therapy. Since tumor burden can affect the response to immunotherapy in murine models,[43] we set up an anti-CTLA4 failure model where anti-CTLA4 was administered on a schedule that was insufficient to inhibit the growth of idMMR neuroblastoma tumors. This model allowed us to determine whether anti-CTLA4 therapy, when not effective, was also inducing terminally exhausted and dysfunctional T cells, similar to anti-PD1 therapy. Therefore, we delayed treatment until idMMR neuroblastoma tumors reached a volume of 400 mm^3. We then administered only two doses of anti-CTLA4 antibody and euthanized the animals 4 days after the last dose (Figure S7A). Anti-CTLA4 therapy did not effectively inhibit tumor growth in this setting (Figure S7B), allowing the examination of the phenotype of T cells in the tumors and spleens of those mice. We did not observe any difference in the frequency of CD8$^+$ and CD4$^+$ T cell among TILs (Figures S7C and S7E) or splenocytes (Figures S7G and S7I) isolated from anti-CTLA4-treated mice. Furthermore, anti-CTLA4 treatment did not induce T cell exhaustion among isolated TILs (Figures S7D and S7F) or T cells from the spleens (Figures S7H and S7J).

The protective effect of anti-CTLA4 treatment against idMMR neuroblastoma tumors is CD4$^+$ T cell dependent and not NK cell dependent

We have shown that anti-CTLA4 treatment cures immunocompetent mice of established idMMR neuroblastoma tumors. It has been proposed that CD4$^+$ T cells are the main effector T cell population for anti-CTLA4 therapy.[39,40] To determine the role of CD4$^+$ T cells on the activity of anti-CTLA4 therapy against idMMR neuroblastoma tumors, we depleted these cells in immunocompetent animals before the treatment of idMMR tumor-bearing mice with anti-CTLA4 antibody (Figure 6A). Depletion of CD4$^+$ T cells abolished the therapeutic effect of anti-CTLA4 therapy against idMMR tumors (Figure 6B), mechanistically confirming the importance of these cells against these immunogenic tumors with low expression of MHC class I- and IFN-related genes.

It has been proposed that, in addition to CD4$^+$ T cells, the anti-tumor effects of anti-CTLA4 treatment depend, to some degree, on natural killer (NK) cell-mediated anti-tumor immunity.[44] We observed lower expression of MHC class I-related genes in idMMR neuroblastoma cells, which could make them susceptible to NK cells.[45] To assess NK cell involvement in the efficacy of anti-CTLA4 treatment against idMMR neuroblastoma tumors, we depleted NK cells in animals, then inoculated them with idMMR neuroblastoma cells and treated them with anti-CTLA4 or isotype control antibodies (Figure S7K). NK cell depletion did not impact the protective effect of anti-CTLA4 therapy against idMMR tumors, nor was there any protective role for NK cells against these tumors in the absence of therapy (Figure S7L).

Anti-CTLA4 therapy broadened anti-tumor immunity in cured hosts previously inoculated with idMMR neuroblastoma tumors

Recurrence is common in high-risk neuroblastoma, and survival following relapse remains poor in these patients. The high recurrence rate in neuroblastoma implies a lack of protective immune memory formation in patients who experience remission followed by recurrence.[46] We next studied the effect of anti-CTLA4 therapy on the formation of immunological memory in cured mice. We rechallenged animals previously cured of idMMR neuroblastoma tumors with the same tumor cells more than 70 days after the initial inoculation (Figure 7A). All previously cured mice rejected idMMR tumors. In contrast, those tumors progressively grew in naive control mice (Figure 7B). These observations indicate that anti-CTLA4 therapy can establish immunological memory in animals cured from idMMR tumors, conferring protection against recurrent neuroblastoma.

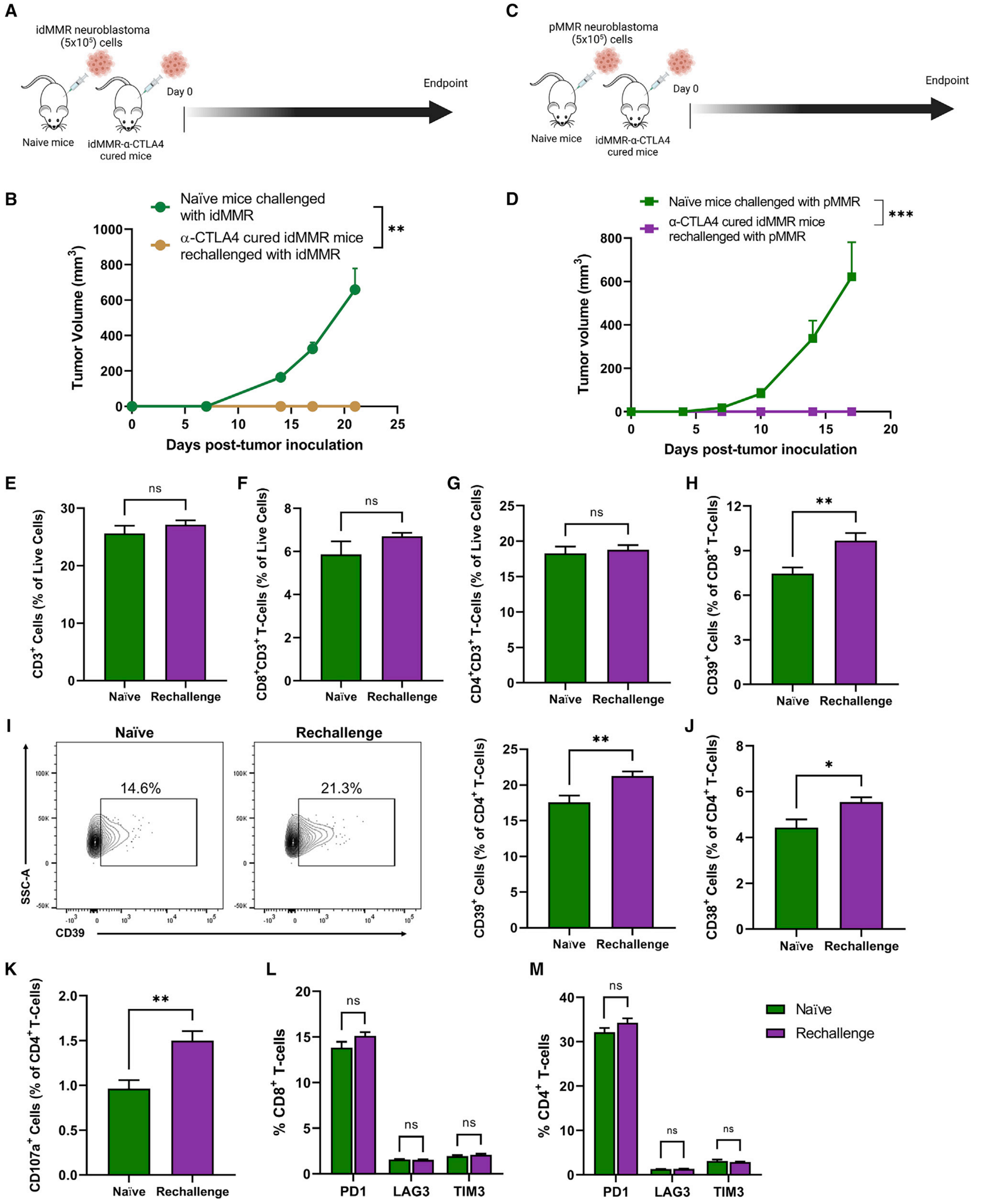

(legend on next page)

Furthermore, it is known that anti-CTLA4 therapy increases T cell receptor (TCR) repertoire diversity among peripheral blood T cells of melanoma and prostate cancer patients.[47] To test whether anti-CTLA4 treatment of idMMR tumors can induce epitope spreading of T cells in cured mice, we inoculated a different cohort of animals, previously cured of idMMR tumors with anti-CTLA4, with the poorly immunogenic pMMR neuroblastoma cells (Figure 7C). We then compared the tumor growth in those mice with tumor growth in naive mice. All mice previously treated with anti-CTLA4 and cured of the immunogenic idMMR tumors rejected the less immunogenic pMMR neuroblastoma tumors (Figure 7D). These findings provide evidence for the induction of epitope spreading in anti-CTLA4-treated mice that can provide protective immunity against the less immunogenic, parental neuroblastoma tumors with an intact MMR pathway.

To mechanistically determine which cells are involved in this phenomenon, we compared the splenic immune profile of animals previously cured of idMMR tumors, then challenged with pMMR cells, with naive mice inoculated with pMMR cells. There was no difference in the overall $CD3^+$, $CD4^+$, or $CD8^+$ T cell levels in the spleens of those mice (Figures 7E–7G). However, we detected an increase in tumor-specific $CD39^+CD8^+$ and $CD39^+CD4^+$ T cells in the spleens of cured animals (Figures 7H, 7I, and S5G). We also observed an increase of activated $CD4^+$ T cells ($CD38^+CD4^+$ T cells) with higher cytotoxic function ($CD107a^+$ $CD4^+$ T cells) in the spleens of previously cured mice that were rechallenged with the poorly immunogenic pMMR neuroblastoma tumor cells (Figures 7J, 7K, and S5H). Notably, anti-CTLA4 treatment did not induce an exhausted phenotype in $CD8^+$ or $CD4^+$ T cells in those cured animals (Figures 7L and 7M), confirming our previous observation in the anti-CTLA4 failure experiment (Figures S7D, S7F, S7H, and S7J).

DISCUSSION

Despite the ongoing success of ICB in the treatment of certain advanced malignancies, neuroblastoma patients still do not benefit from these drugs. A common characteristic of solid adult tumors with a higher response rate to ICB is higher TMB linked to the production of more neoantigens.[41] However, neuroblastoma has a low TMB background, does not show an MMR-deficient or MSI-h phenotype, and is poorly immunogenic,[48] making it a poor candidate for single or combination ICB treatment. Therefore, strategies to transform neuroblastoma into an immunogenic cancer are of high clinical interest to identify more effective combination therapies involving ICBs.

We showed that high-risk neuroblastoma patients with $CD8^+$ T cell-infiltrated tumors and memory T cell-associated markers had improved survival. These findings suggest that increased T cell immunity and T cell memory responses in high-risk neuroblastoma tumors can be a favorable prognostic marker, regardless of the *MYCN* status of tumors. Therefore, strategies to enhance T cell infiltration into neuroblastoma tumors and increase T cell memory toward neuroblastoma tumors are of potential therapeutic value in this immune-cold cancer. We also showed, for the first time, that by knocking out the *MLH1* gene and inducing MMR deficiency in neuroblastoma tumors they became more immunogenic and highly infiltrated by effector cytotoxic $CD8^+$ and $CD4^+$ T cells, along with an increase in $CD39^+CD8^+$ tumor-specific T cells. The high tumor reactivity of $CD39^+CD8^+$ T cells is essential for tumor control in both primary and metastatic lesions in various cancers.[25,26] These findings indicate that targeting the DNA MMR pathway can be a therapeutic strategy to increase the immunogenicity of neuroblastoma and potentially improve patient prognosis.

For the first time, we also showed that anti-CTLA4 therapy cures most of the animals with idMMR neuroblastoma tumors and induces a long-term immune memory response with epitope spreading that allows for a highly effective anti-tumor response against both idMMR and pMMR neuroblastoma tumors upon rechallenge. This effect is mainly $CD4^+$ T cell mediated, as depletion of these cells abolished the therapeutic effect of anti-CTLA4 against idMMR neuroblastoma tumors. Moreover, induced MMR deficiency decreases MHC class I-related and *B2M* gene expression in neuroblastoma cells, rendering them less sensitive to $CD8^+$ T cell cytotoxicity and potentially anti-PD1 therapy. Indeed, we confirmed the lack of treatment benefit from anti-PD1 treatment against idMMR neuroblastoma tumors, which mainly relies on $CD8^+$ T cells for efficacy. Moreover, anti-PD1 therapy against idMMR neuroblastoma tumors induced T cell exhaustion among TILs, similar to a report in lung cancer patients with MHC class I downregulated tumors.[34] This effect of anti-PD1 therapy against the immunogenic but low MHC class I-expressing idMMR tumors shows that inducing MMR deficiency would not universally sensitize tumors to ICBs, particularly low TMB and immune-cold neuroblastoma tumors.

Impairing DNA repair pathways is commonly used therapeutically in cancer patients, and blocking the MMR pathway has been proposed as a new strategy to sensitize adult tumors to ICB treatment.[12] A future therapeutic strategy for inducing MMR deficiency in tumors

Figure 7. Inducing MMR deficiency in combination with anti-CTLA4 treatment stimulates broad anti-tumor immune memory response

(A–M) Cured mice from the idMMR neuroblastoma, anti-CTLA4 treated group (n = 4) inoculated with idMMR neuroblastoma cells (5×10^5) at around 70 days later and compared with naive mice (n = 4) similarly inoculated with 8-week idMMR neuroblastoma cells (A). Tumor growth of rechallenged and naive mice (B). Cured mice from the idMMR neuroblastoma, anti-CTLA4 treated group (n = 6) inoculated with 8-week pMMR neuroblastoma cells (5×10^5) approximately 60 days later and compared with naive mice (n = 8) similarly inoculated with 8-week pMMR cells (C). Tumor growth of naive and rechallenged mice (D). Spleens were harvest 18 days after rechallenging with pMMR cells and stained for flow cytometry analysis of T cells. Data collected are presented as the percentage of live cells, $CD3^+$ (E), $CD8^+CD3^+$ (F), and $CD4^+CD3^+$ (G) T cells. Quantification of $CD39^+$ cells as a percentage of $CD8^+$ T cells (H) and $CD39^+$ (I), $CD38^+$ (J), and $CD107a^+$ (K) cells as a percentage of $CD4^+$ T cells. Quantification of $PD1^+$, $LAG3^+$, $TIM3^+$ as a percentage of $CD8^+$ and $CD4^+$ T cells (L and M). See also Figure S7. Statistical analyses were performed using the unpaired two-tailed Student's t test. *p $\leq$ 0.05, **p $\leq$ 0.01; ns (not significant), error bars indicate SEM.

will likely involve small-molecule inhibitors and systemic inhibition of the MMR pathway. However, blocking the MMR pathway in a growing child with neuroblastoma over an extended period would not be feasible. Therefore, we show that even treatment over a relatively short time window (8 weeks) to accumulate enough immunogenic mutations in neuroblastoma tumors is sufficient to induce T cell infiltration into these poorly immunogenic tumors and sensitizes them to anti-CTLA4 treatment. In addition, we have previously shown that cancer cells are more sensitive to the toxic effects of blockade of DNA repair mechanisms than normal cells.[49,50] This is most likely due to higher levels of mutations in cancer cells than in normal cells and the enhanced capacity for normal cells to repair DNA damage making this approach feasible, at least for a short duration of time in children.

From an immunological perspective, inducing MMR deficiency can produce more neoantigens in tumor cells.[11] CTLA4 attenuates TCR signaling strength and inhibits early T cell activation.[40] Therefore, blocking CTLA4 in the context of inducing MMR deficiency in tumors can allow subdominant epitopes in those tumors to generate high-affinity T cell clones able to recognize even cryptic epitopes on tumor cells.[51] Furthermore, anti-CTLA4 therapy induces a robust $CD4^+$-based T cell response in patients,[52] similar to our observation in cured mice rechallenged with pMMR tumors. We also confirmed that the anti-CTLA4 effect was mainly driven by $CD4^+$ T cells and did not find any role for NK cells in the efficacy of anti-CTLA4 treatment against idMMR neuroblastoma tumors. These findings further confirm the protective role of T cells against idMMR neuroblastoma tumors despite their lower MHC class I gene expression, highlighting the importance of this T cell-based treatment strategy in neuroblastoma.

Furthermore, animals cured by anti-CTLA4 therapy from idMMR tumors had an increase in tumor-reactive T cells ($CD39^+CD8^+$ and $CD39^+CD4^+$ T cells) and activated $CD4^+$ T cells ($CD38^+CD4^+$ T cells) with higher cytotoxic potential ($CD107a^+CD4^+$ T cells) in their spleens after rechallenging with the pMMR neuroblastoma tumors. The activation of $CD4^+$ T cells in the context of epitope spreading and immune memory is critical as these helper cells can also support the functionality of $CD8^+$ T cells. Importantly, our findings indicate that anti-CTLA4 treatment of idMMR neuroblastoma tumors can induce a robust T cell response involving $CD8^+$ and $CD4^+$ T cells. This has important effects on long-term immune memory and epitope spreading, which is crucial for a potential treatment strategy in relapse-prone high-risk neuroblastoma patients. In the context of developing novel treatment strategies for neuroblastoma, these are important findings as recurrence of disease is common in high-risk patients following the standard treatment, which indicates the lack of protective T cell immunity. Thus, strategies involving inducing MMR deficiency and anti-CTLA4 therapy may prevent disease recurrence following complete response in children. One limitation of our findings is that the neuroblastoma tumors were grown subcutaneously as a proof of principle. Heterotopic tumor models often lack the complexity of the original anatomical position of the tumor and its immune microenvironment.[53] However, given the aggressive nature of the neuro-2a neuroblastoma tumor model and how effective anti-CTLA4 treatment has been against idMMR neuroblastoma tumors, we anticipate a potentially effective response against orthotopic neuroblastoma tumors in renal capsules or metastatic disease in the bone marrow. Future studies are warranted to address these important questions.

The FDA has approved pembrolizumab for first-line treatment of colorectal cancer patients with MMR deficiency or MSI-h tumors and in second line for any tumor type with MSI-h status or MMR deficiency in adults and children.[54,55] However, despite a higher TMB presence in these tumors, less than 40% of these patients respond to anti-PD1 therapy.[55] The lack of clinical benefit from anti-PD1 treatment in a proportion of patients with these types of tumors may be driven by the negative effect of treatment on patient T cells, potentially due to lower MHC class I levels in these tumors. Our findings indicate that anti-PD1 therapy uniquely induces the expression of inhibitory receptors on TILs when used against neuroblastoma tumors with induced MMR deficiency, but this is not the case with anti-CTLA4 therapy. Therefore, anti-CTLA4 therapy may offer an alternative to anti-PD1 therapy in patients with MSI-h tumors, particularly those with a low TMB background. Our findings also show important anti-PD1 resistance mechanisms in idMMR neuroblastoma tumors, which have not been shown in other dMMR adult cancers.

Our results can help justify the development of small-molecule inhibitors targeting the MMR pathway and combination treatment with anti-CTLA4 in neuroblastoma patients. In summary, our findings suggest that inducing MMR deficiency in neuroblastoma tumors combined with anti-CTLA4 therapy may serve as a novel potential T cell-based treatment strategy for treating high-risk neuroblastoma.

MATERIALS AND METHODS CELL LLINES

The murine neuroblastoma cell line, neuro-2a cell line, was obtained from the American Type Culture Collection (ATCC) and was used for the experiments described below (Table S1). Neuroblastoma cells were tested to ensure viability and lack of contaminating mycoplasma and common viruses (Idexx Bioresearch IMPACT II RADIL testing, Columbia, MO). Cells were cultured in RPMI 1640 media (Wisent Bio Products, Saint-Jean Baptise, QC) supplemented with 10% heat-inactivated fetal bovine serum (FBS) (ThermoFisher Scientific, Waltham, MA, USA) at 37 °C, 5% CO_2. For most of the experiments, cells were expanded to a ~70-80% confluency, then washed with PBS (Wisent Bio Products, Saint-Jean Baptise, QC, CA) before harvesting cells with trypsin (Wisent Bio Products, Saint-Jean Baptise, QC, CA).

Generating neuroblastoma MLH1 knockout cell line

Clustered Regularly Interspaced Short Palindromic Repeats (CRISPR) and the CRISPR-associated protein (Cas9) were used to degrade target genetic material to knockout a gene of interest. We used an *MLH1* CRISPR Cas9 KO plasmid (Santa Cruz Biotechnology, Dallas, TX, USA) in order to knockout the *MLH1* gene in the neuro-2a cell line.

This CRISPR/Cas9 plasmid consists of a pool of 3 different plasmids with distinct target-specific guide RNAs, a GFP encoding region, and a Cas9 encoding region. A pool of 3 plasmids targeting different portions of the *MLH1* gene was used in order to maximize knockout efficiency. Sequences of the three guide RNAs are as follows, sc-421660 A – sense: TACCTCACCACGAAAGCCAT, sc-421660 B – sense: TCAC CGTGATCAGGGTGCCC, and sc-421660 C – sense: ACTTACGGT TGATGAAGAGT. Other important regions of the plasmids are indicated on the Santa Cruz Biotechnology website (https://www.scbt.com/p/mlh1-crispr-knockout-and-activation-products-m).

In a 6-well plate, 50,000 neuroblastoma cells were seeded in 3 ml of RPMI 1640 medium with 10% FBS and grown to 80% confluency. After 24 hours, 1 μg of plasmid DNA was mixed with 3 ml of lipofectamine 3000 (ThermoFisher Scientific, Waltham, MA, USA) and 500 μl of the medium. Cells were incubated for 24 hours at 37 °C, 5% CO_2. Successful plasmid transfection was visualized using fluorescence-activated cell sorting (FACS) for green fluorescence protein (GFP) fluorescence as transfected cells transiently expressed GFP. GFP^+ cells were sorted, and one cell was seeded per well in a 96-well plate. Cells were grown until a colony was formed, then trypsin was used as previously described. Cells were expanded to a final culture in T75 flasks. Successful and failed KO of each cell line were cultured for use in methods described below.

Mice

Female A/J mice were purchased from the Jackson Laboratory (Jackson Laboratory, Bar Habor, ME, USA), and immunodeficient SHO mice were purchased from Charles River (Charles River, Wilmington, MA, USA). Mice were all 6-8 weeks of age when acquired. These mice were housed at the Victoria Research Lab Vivarium. All animal experiments were approved by the Animal Care Committee (ACC) at Western University.

In vivo tumor models

For *in vivo* experiments, $5x10^5$ pMMR or idMMR neuroblastoma cells were inoculated subcutaneously (s.c.) into A/J mice (day 0). Tumors were palpable ∼10 days post tumor inoculation. At day10, depending on the experiment, mice were given 250 μg of anti-PD1 antibody (clone RMP1-14, BioXCell), 200 μg of anti-CTLA4 antibody (clone 9H10, BioXCell) or a combination of both in 100 μl of PBS injected intraperitoneally (i.p.) every 3 days for a total of 3 injections on days 10, 13, and 16. Control mice received 250 μg of Rat IgG2a (clone 2A3, BioXCell) for the anti-PD1 isotype control or 200 μg of Syrian Hamster IgG (BioXCell) for the anti-CTLA4 isotype control, or a combination of both. For the anti-CTLA4 failure model, mice were treated with anti-CTLA4 or isotype control, as described above, on days 14 and 17 after tumor inoculation. Tumor volumes were measured using calipers and calculated by measuring their length and width and recorded in mm^3. When the first tumor(s) volumes reached ∼$1400mm^3$, all mice were euthanized.

When analyzing tumor growth in immunodeficient animals, $5x10^5$ pMMR (C12) and idMMR (C4) neuroblastoma cells were inoculated s.c. into SHO mice (day 0). Tumor volumes were measured over the course of the experiment, and mice were sacrificed individually when their tumor volumes reached ∼1400 mm^3.

For $CD4^+$ T-cell depletion experiments, mice were injected i.p with 100 ug of anti-CD4 (clone GK1.5, BioXCell) or PBS for control. Mice were treated with anti-CD4 or PBS 24 hours before the inoculation of 5 $x10^5$ idMMR neuroblastoma cells (8 weeks) s.c. followed by additional doses on days 3, 7, and 11 after tumor inoculation for a total of 4 doses. Mice were treated with isotype control antibody (200 ug) or anti-CTLA4 (200 ug) on days 10, 13, 16 after tumor inoculation. Tumor volumes were measured, and mice were sacrificed when first tumor(s) reached ∼1400 $mm^{3.}$

For NK-cell depletion, mice were injected i.p. with 100 μl of anti-asialo GM1 antibody (Wako Chemicals, Richmond, VA, USA) at 4 mg/ml or PBS, 24 hours and 1 hour before tumor cell inoculation (day 0) and subsequently every 7 days until day 21 for a total of 5 injections. Treatment with anti-CTLA4 or isotype control was administered on days 10, 13, and 16, as described above. Mice were sacrificed individually when their tumor volumes reached ∼1400 mm^3.

For the rechallenge experiments, mice that had idMMR neuroblastoma tumors, which were cured by anti-CTLA4 treatment, or naïve age-matched mice were inoculated s.c. with $5x10^5$ neuro-2a cells into the left flank. In separate experiments, mice were rechallenged with idMMR or pMMR neuroblastoma cells 75 or 61 days after the initial tumor injection, respectively. Once the average tumor volume of naïve mice reached ∼650 mm^3, all mice were sacrificed.

Flow cytometry

Tumors were harvested and single-cell suspensions were obtained with the mouse tumor dissociation kit according to manufacturer's instructions (Miltenyi Biotec, USA). Red blood cells in tumor and spleen cell suspensions were lysed with ACK lysis buffer. Cells were resuspended in 5% FBS in PBS and $1x10^6$ cells were stained with a Zombie viability dye for 20 minutes at room temperature and then incubated with CD16/32 for 10 minutes on ice to block Fc receptors. Cells were then incubated with a cocktail of fluorochrome-conjugated antibodies (Biolegend, USA) (Table S1) for 20 minutes on ice for cell surface staining. Cells were fixed with 2% paraformaldehyde for 20 minutes at room temperature for storage. Data was acquired with the BD LSRII flow cytometer (BD, USA) and analyzed with FlowJo (BD, USA) (Figure S5D).

IFN-β enzyme-linked immunosorbent assay

8- and 14-week pMMR and idMMR neuroblastoma cells were cultured for 48 h, and the supernatant was removed when confluency reached ∼90%. The supernatant was centrifuged at 14,000 RPM to remove cellular debris, and IFN-β was quantified using the mouse IFN-β ELISA Kit (PBL Assay Science) according to the manufacturer's instructions.

RNA sequencing and transcriptomic analysis

RNA from pMMR and idMMR neuroblastoma cells cultured for 8 weeks and 14 weeks was extracted using the RNeasy mini kit (Qiagen, USA). RNA sequencing was conducted by the London Regional Genomics Center (Robarts Research Institute, London, ON.) with the Illumina NextSeq High Output 75 Cycle Sequencing Kit and Illumina NextSeq sequencer. Fastq reads were mapped to the genome using STAR, and subsequent FPKM read normalization and differential gene expression were obtained using HOMER package.

Mining TCGA, target, and GSE49710 datasets

The Cancer Genome Atlas (TCGA) database was bioinformatically mined to the colorectal adenocarcinoma (COAD READ) dataset. mRNA sequencing data was obtained from the Broad Genome Data Analysis Firehose database (https://gdac.broadinstitute.org/). This dataset was level 3 RSEM (RNA-Seq by Expectation Maximization) normalized illumina high throughput sequencing data. The normalized RNA seq data file name is "illuminahiseq_rnaseqv2-RSEM_genes_normalized (MD5)" and was subsequently converted into a CSV file format. The COAD READ cohort has 631 total samples. Using Python libraries (csv, os and itertools), 3 excel files were created with patient ID information for each sample along with RNA-seq values for a number of genes. Samples were divided into the 3 excel files according to whether they were classified as "normal", “MSS” or “MSI-h” in the clinical file titled “Merge_Clinical (MD5)” on the Firehose database website (https://gdac.broadinstitute.org/). COAD READ patient samples with both microsatellite status information along with gene expression data were therefore grouped into normal (n=51), MSS (n=257) and MSI-h (n=51) primary tumor tissue samples. Metastatic samples were not included in the analysis. Using the python library Seaborn, box plots were generated in order to compare gene expression between groups. The center line of boxplots represents the media and the upper and lower limits indicate the 75th and 25th percentiles respectively. Mann-Whitney U test was used to statistically compare the groups. In order to generate the overall survival graphs, we analyzed gene expression as described above. Moreover, we stratified patients according to their gene expression into “high” and “low” groups and used the lifelines library to generate the overall survival plots. The 2-sided log-rank test was used to compare the overall survival in patients highly expressing a gene and patients expressing the gene to a lower extent.

Similarly, the *Therapeutically Applicable Research to Generate Effective Treatments* (TARGET) database was computationally analyzed to assess the importance of gene expression in the context of the overall survival of patients. High-risk and low-risk patient survival was obtained from the National Cancer Institute, Office of Cancer Genomics website https://ocg.cancer.gov/programs/target/data-matrix under the “Clinical File”. Survival was plotted using the lifelines Python library and significance was determined using the 2-sided log-rank test. High-risk patients were further stratified according to mRNA expression of multiple genes. Sequencing was conducted using Illumina Genome Analyzer IIx or Hi-Seq 2000. mRNA sequencing data was obtained from the mRNA seq column under the “DCC Open” tab. RNAseq reads were pre-standardized to fragments per kilobase of transcript per million of mapped reads (FPKM). Overall survival of high-risk patients was stratified according to upper and lower 30th percentile expression of a number of genes. Similar analysis was completed in *MYCN* amplified and *MYCN* non-amplified patients, stratifying samples obtained from patients according to gene expression as described above. Survival was plotted using lifelines library and the 2-sided log-rank test was used to compare the overall survival in high-risk patients highly and minimally expressing a given gene.

The GSE49710 microarray dataset was bioinformatically mined to study neuroblastoma transcriptomic profiles of 322 low-risk and 176 high-risk tumor samples. Normalized gene expression of the GSE49710 microarray dataset was obtained from GEO (https://www.ncbi.nlm.nih.gov/geo/query/acc.cgi?acc=GSE49710) and expression levels of key immune related genes was analyzed comparing low-risk and high-risk tumor samples.

Statistical Analysis

In vitro data are represented as mean ± standard deviation (SD). Results were analyzed using Student’s two-tailed unpaired t-test. Moreover, *in vivo* experiments requiring the comparison between more than two groups, statistical analysis was performed using the one-way ANOVA test. These results are represented as mean ± standard error of the mean (SEM). Finally, for the survival experiments, statistical analysis was performed using the two-sided log-rank (Mantel-Cox) test. For all tests, a p-value < 0.05 (indicated by an asterisk [*] in graphed data) was chosen *a priori* as an indication that the null hypothesis could be rejected. However, data showing differences at more stringent bars of significance (** $p \leq 0.01$, *** $p \leq 0.001$, **** $p \leq 0.0001$) are also shown as a matter of interest only: where significance is ascribed, only $p \leq 0.05$ was considered. Where p values were greater than 0.05, “ns” indicates “not significant”.

CONSENT FOR PUBLICATION

All authors have approved the manuscript for publication.

AVAILABILITY OF DATA AND MATERIAL

Data and material used in this study are available on reasonable request. The data used for the bioinformatics analysis are available at https://portal.gdc.cancer.gov/projects.

COMPETING INTERESTS

Authors declare no competing interest.

SUPPLEMENTAL INFORMATION

Supplemental information can be found online at https://doi.org/10.1016/j.ymthe.2022.08.025.

ACKNOWLEDGMENTS

The results published here are in part based upon data generated by the TCGA Research Network: https://www.cancer.gov/tcga and by the Therapeutically Applicable Research to Generate Effective Treatments

(https://ocg.cancer.gov/programs/target) initiative, phs000218. Schematics were created with BioRender.com (2022). Funding was provided by a Canadian Institutes of Health Research grant MOP#389137 (to S.M.V., J.K., and X.Z.), and a London Regional Cancer Program's Catalyst Grant Program, Keith Samitt Translational Research grant (to S.M.V.).

AUTHOR CONTRIBUTIONS

M.E.-H., L.G., X.Z., J.K., and S.M.V. conceptualized the study and planned the experiments. M.E.-H., L.G., M.MYH., M.K., and R.F. performed all experiments. M.E.-H., L.G., M.MYH., and M.K. analyzed the data. M.E.-H., L.G., M.MYH., and S.M.V. wrote and reviewed the paper with advice from other authors. S.M.V. supervised the work.

DECLARATION OF INTERESTS

The authors declare no competing interests.

REFERENCES

1. Sokol, E., and Desai, A.V. (2019). The evolution of risk classification for neuroblastoma. Children (Basel) *6*, E27.
2. Wu, X., Nelson, M., Basu, M., Srinivasan, P., Lazarski, C., Zhang, P., Zheng, P., and Sandler, A.D. (2021). MYC oncogene is associated with suppression of tumor immunity and targeting Myc induces tumor cell immunogenicity for therapeutic whole cell vaccination. J. Immunother. Cancer *9*, e001388.
3. Dzieran, J., Rodriguez Garcia, A., Westermark, U.K., Henley, A.B., Eyre Sánchez, E., Träger, C., Johansson, H.J., Lehtiö, J., and Arsenian-Henriksson, M. (2018). MYCN-amplified neuroblastoma maintains an aggressive and undifferentiated phenotype by deregulation of estrogen and NGF signaling. Proc. Natl. Acad. Sci. USA *115*, E1229–E1238.
4. Keyel, M.E., and Reynolds, C.P. (2019). Spotlight on dinutuximab in the treatment of high-risk neuroblastoma: development and place in therapy. Biologics *13*, 1–12.
5. Waldman, A.D., Fritz, J.M., and Lenardo, M.J. (2020). A guide to cancer immunotherapy: from T cell basic science to clinical practice. Nat. Rev. Immunol. *20*, 651–668.
6. Voeller, J., and Sondel, P.M. (2019). Advances in anti-GD2 immunotherapy for treatment of high-risk neuroblastoma. J. Pediatr. Hematol. Oncol. *41*, 163–169.
7. Straathof, K., Flutter, B., Wallace, R., Jain, N., Loka, T., Depani, S., Wright, G., Thomas, S., Cheung, G.W.K., Gileadi, T., et al. (2020). Antitumor activity without on-target off-tumor toxicity of GD2-chimeric antigen receptor T cells in patients with neuroblastoma. Sci. Transl. Med. *12*, eabd6169.
8. Robert, C. (2020). A decade of immune-checkpoint inhibitors in cancer therapy. Nat. Commun. *11*, 3801.
9. Lu, C., Guan, J., Lu, S., Jin, Q., Rousseau, B., Lu, T., Stephens, D., Zhang, H., Zhu, J., Yang, M., et al. (2021). DNA sensing in mismatch repair-deficient tumor cells is essential for anti-tumor immunity. Cancer Cell *39*, 96–108.e6.
10. Le, D.T., Uram, J.N., Wang, H., Bartlett, B.R., Kemberling, H., Eyring, A.D., Skora, A.D., Luber, B.S., Azad, N.S., Laheru, D., et al. (2015). PD-1 blockade in tumors with mismatch-repair deficiency. N. Engl. J. Med. *372*, 2509–2520.
11. Germano, G., Lamba, S., Rospo, G., Barault, L., Magrì, A., Maione, F., Russo, M., Crisafulli, G., Bartolini, A., Lerda, G., et al. (2017). Inactivation of DNA repair triggers neoantigen generation and impairs tumour growth. Nature *552*, 116–120.
12. Truini, A., Germano, G., and Bardelli, A. (2018). Inactivation of DNA repair-prospects for boosting cancer immune surveillance. Genome Med. *10*, 91.
13. Reilly, N.M., Novara, L., Di Nicolantonio, F., and Bardelli, A. (2019). Exploiting DNA repair defects in colorectal cancer. Mol. Oncol. *13*, 681–700.
14. Southgate, H.E.D., Chen, L., Curtin, N.J., and Tweddle, D.A. (2020). Targeting the DNA damage response for the treatment of high risk neuroblastoma. Front. Oncol. *10*, 371.
15. Spolski, R., Li, P., and Leonard, W.J. (2018). Biology and regulation of IL-2: from molecular mechanisms to human therapy. Nat. Rev. Immunol. *18*, 648–659.
16. Hope, J.L., Stairiker, C.J., Bae, E.A., Otero, D.C., and Bradley, L.M. (2019). Striking a balance-cellular and molecular drivers of memory T cell development and responses to chronic stimulation. Front. Immunol. *10*, 1595.
17. Lin, A., Zhang, J., and Luo, P. (2020). Crosstalk between the MSI status and tumor microenvironment in colorectal cancer. Front. Immunol. *11*, 2039.
18. Kloor, M., and von Knebel Doeberitz, M. (2016). The immune biology of microsatellite-unstable cancer. Trends Cancer *2*, 121–133.
19. Cheng, X., Cao, Y., Wang, X., Cheng, L., Liu, Y., Lei, J., Peng, W., and Shi, D. (2022). Systematic pan-cancer analysis of KLRB1 with prognostic value and immunological activity across human tumors. J. Immunol. Res. *2022*, 5254911.
20. Wang, J., Zhang, X., Li, J., Ma, X., Feng, F., Liu, L., Wu, J., and Sun, C. (2020). ADRB1 was identified as a potential biomarker for breast cancer by the co-analysis of tumor mutational burden and immune infiltration. Aging (Albany NY) *13*, 351–363.
21. Zhang, W., Ge, Y., Cheng, Q., Zhang, Q., Fang, L., and Zheng, J. (2018). Decorin is a pivotal effector in the extracellular matrix and tumour microenvironment. Oncotarget *9*, 5480–5491.
22. Guillory, B., Sakwe, A.M., Saria, M., Thompson, P., Adhiambo, C., Koumangoye, R., Ballard, B., Binhazim, A., Cone, C., Jahanen-Dechent, W., and Ochieng, J. (2010). Lack of fetuin-A (alpha2-HS-glycoprotein) reduces mammary tumor incidence and prolongs tumor latency via the transforming growth factor-beta signaling pathway in a mouse model of breast cancer. Am. J. Pathol. *177*, 2635–2644.
23. Yang, Y., Gao, L., Weng, N.N., Li, J.J., Liu, J.L., Zhou, Y., Liao, R., Xiong, Q.L., Xu, Y.F., Varela-Ramirez, A., and Zhu, Q. (2021). Identification of novel molecular therapeutic targets and their potential prognostic biomarkers among kinesin superfamily of proteins in pancreatic ductal adenocarcinoma. Front. Oncol. *11*, 708900.
24. Yasuoka, H., Yamaguchi, Y., and Feghali-Bostwick, C.A. (2009). The pro-fibrotic factor IGFBP-5 induces lung fibroblast and mononuclear cell migration. Am. J. Respir. Cell Mol. Biol. *41*, 179–188.
25. Zheng, Y., Meng, X., Zweigenbaum, P., Chen, L., and Xia, J. (2020). Hybrid phenotype mining method for investigating off-target protein and underlying side effects of anti-tumor immunotherapy. BMC Med. Inform. Decis. Mak. *20* (*Suppl 3*), 133.
26. Pan, J.H., Zhou, H., Cooper, L., Huang, J.L., Zhu, S.B., Zhao, X.X., Ding, H., Pan, Y.L., and Rong, L. (2019). LAYN is a prognostic biomarker and correlated with immune infiltrates in gastric and colon cancers. Front. Immunol. *10*, 6.
27. Shin, J.H., Moreno-Nieves, U.Y., Zhang, L.H., Chen, C., Dixon, A.L., Linde, M.H., Mace, E.M., and Sunwoo, J.B. (2021). AHR regulates NK cell migration via ASB2-mediated ubiquitination of filamin A. Front. Immunol. *12*, 624284.
28. Aktas, E., Kucuksezer, U.C., Bilgic, S., Erten, G., and Deniz, G. (2009). Relationship between CD107a expression and cytotoxic activity. Cell. Immunol. *254*, 149–154.
29. Duhen, T., Duhen, R., Montler, R., Moses, J., Moudgil, T., de Miranda, N.F., Goodall, C.P., Blair, T.C., Fox, B.A., McDermott, J.E., et al. (2018). Co-expression of CD39 and CD103 identifies tumor-reactive CD8 T cells in human solid tumors. Nat. Commun. *9*, 2724.
30. Tallón de Lara, P., Castañón, H., Vermeer, M., Núñez, N., Silina, K., Sobottka, B., Urdinez, J., Cecconi, V., Yagita, H., Movahedian Attar, F., et al. (2021). CD39(+) PD-1(+)CD8(+) T cells mediate metastatic dormancy in breast cancer. Nat. Commun. *12*, 769.
31. Wölfl, M., Jungbluth, A.A., Garrido, F., Cabrera, T., Meyen-Southard, S., Spitz, R., Ernestus, K., and Berthold, F. (2005). Expression of MHC class I, MHC class II, and cancer germline antigens in neuroblastoma. Cancer Immunol. Immunother. *54*, 400–406.
32. Janikovits, J., Müller, M., Krzykalla, J., Körner, S., Echterdiek, F., Lahrmann, B., Grabe, N., Schneider, M., Benner, A., Doeberitz, M.v.K., and Kloor, M. (2018). High numbers of PDCD1 (PD-1)-positive T cells and B2M mutations in microsatellite-unstable colorectal cancer. Oncoimmunology *7*, e1390640.
33. Ijsselsteijn, M.E., Petitprez, F., Lacroix, L., Ruano, D., van der Breggen, R., Julie, C., Morreau, H., Sautès-Fridman, C., Fridman, W.H., and de Miranda, N.F.d.C.C. (2019). Revisiting immune escape in colorectal cancer in the era of immunotherapy. Br. J. Cancer *120*, 815–818.
34. Gettinger, S., Choi, J., Hastings, K., Truini, A., Datar, I., Sowell, R., Wurtz, A., Dong, W., Cai, G., Melnick, M.A., et al. (2017). Impaired HLA class I antigen processing and

presentation as a mechanism of acquired resistance to immune checkpoint inhibitors in lung cancer. Cancer Discov. *7*, 1420–1435.

35. Romieu-Mourez, R., François, M., Boivin, M.N., Stagg, J., and Galipeau, J. (2007). Regulation of MHC class II expression and antigen processing in murine and human mesenchymal stromal cells by IFN-gamma, TGF-beta, and cell density. J. Immunol. *179*, 1549–1558.

36. Zhou, F. (2009). Molecular mechanisms of IFN-gamma to up-regulate MHC class I antigen processing and presentation. Int. Rev. Immunol. *28*, 239–260.

37. Grasso, C.S., Giannakis, M., Wells, D.K., Hamada, T., Mu, X.J., Quist, M., Nowak, J.A., Nishihara, R., Qian, Z.R., Inamura, K., et al. (2018). Genetic mechanisms of immune evasion in colorectal cancer. Cancer Discov. *8*, 730–749.

38. Germano, G., Lu, S., Rospo, G., Lamba, S., Rousseau, B., Fanelli, S., Stenech, D., Le, D.T., Hays, J., Totaro, M.G., et al. (2021). CD4 T cell-dependent rejection of beta-2 microglobulin null mismatch repair-deficient tumors. Cancer Discov. *11*, 1844–1859.

39. Wei, S.C., Levine, J.H., Cogdill, A.P., Zhao, Y., Anang, N.A.A.S., Andrews, M.C., Sharma, P., Wang, J., Wargo, J.A., Pe'er, D., and Allison, J.P. (2017). Distinct cellular mechanisms underlie anti-CTLA-4 and anti-PD-1 checkpoint blockade. Cell *170*, 1120–1133.e17.

40. Hong, M.M.Y., and Maleki Vareki, S. (2022). Addressing the elephant in the immunotherapy room: effector T-cell priming versus depletion of regulatory T-cells by anti-CTLA-4 therapy. Cancers (Basel) *14*, 1580.

41. Maleki Vareki, S. (2018). High and low mutational burden tumors versus immunologically hot and cold tumors and response to immune checkpoint inhibitors. J. Immunother. Cancer *6*, 157.

42. Mandal, R., Samstein, R.M., Lee, K.W., Havel, J.J., Wang, H., Krishna, C., Sabio, E.Y., Makarov, V., Kuo, F., Blecua, P., et al. (2019). Genetic diversity of tumors with mismatch repair deficiency influences anti-PD-1 immunotherapy response. Science *364*, 485–491.

43. Taylor, M.A., Hughes, A.M., Walton, J., Coenen-Stass, A.M.L., Magiera, L., Mooney, L., Bell, S., Staniszewska, A.D., Sandin, L.C., Barry, S.T., et al. (2019). Longitudinal immune characterization of syngeneic tumor models to enable model selection for immune oncology drug discovery. J. Immunother. Cancer *7*, 328.

44. Sanseviero, E., O'Brien, E.M., Karras, J.R., Shabaneh, T.B., Aksoy, B.A., Xu, W., Zheng, C., Yin, X., Xu, X., Karakousis, G.C., et al. (2019). Anti-CTLA-4 activates intratumoral NK cells and combined with IL15/IL15Rα complexes enhances tumor control. Cancer Immunol. Res. *7*, 1371–1380.

45. Paul, S., and Lal, G. (2017). The molecular mechanism of natural killer cells function and its importance in cancer immunotherapy. Front. Immunol. *8*, 1124.

46. Liu, C., Somasundaram, A., Manne, S., Gocher, A.M., Szymczak-Workman, A.L., Vignali, K.M., Scott, E.N., Normolle, D.P., John Wherry, E., Lipson, E.J., et al. (2020). Neuropilin-1 is a T cell memory checkpoint limiting long-term antitumor immunity. Nat. Immunol. *21*, 1010–1021.

47. Cha, E., Klinger, M., Hou, Y., Cummings, C., Ribas, A., Faham, M., and Fong, L. (2014). Improved survival with T cell clonotype stability after anti-CTLA-4 treatment in cancer patients. Sci. Transl. Med. *6*, 238ra70.

48. Prigione, I., Corrias, M.V., Airoldi, I., Raffaghello, L., Morandi, F., Bocca, P., Cocco, C., Ferrone, S., and Pistoia, V. (2004). Immunogenicity of human neuroblastoma. Ann. N. Y. Acad. Sci. *1028*, 69–80.

49. Rytelewski, M., Maleki Vareki, S., Mangala, L.S., Romanow, L., Jiang, D., Pradeep, S., Rodriguez-Aguayo, C., Lopez-Berestein, G., Figueredo, R., Ferguson, P.J., et al. (2016). Reciprocal positive selection for weakness - preventing olaparib resistance by inhibiting BRCA2. Oncotarget *7*, 20825–20839.

50. Rytelewski, M., Tong, J.G., Buensuceso, A., Leong, H.S., Maleki Vareki, S., Figueredo, R., Di Cresce, C., Wu, S.Y., Herbrich, S.M., Baggerly, K.A., et al. (2014). BRCA2 inhibition enhances cisplatin-mediated alterations in tumor cell proliferation, metabolism, and metastasis. Mol. Oncol. *8*, 1429–1440.

51. Brossart, P. (2020). The role of antigen spreading in the efficacy of immunotherapies. Clin. Cancer Res. *26*, 4442–4447.

52. Ng Tang, D., Shen, Y., Sun, J., Wen, S., Wolchok, J.D., Yuan, J., Allison, J.P., and Sharma, P. (2013). Increased frequency of ICOS+ CD4 T cells as a pharmacodynamic biomarker for anti-CTLA-4 therapy. Cancer Immunol. Res. *1*, 229–234.

53. Ireson, C.R., Alavijeh, M.S., Palmer, A.M., Fowler, E.R., and Jones, H.J. (2019). The role of mouse tumour models in the discovery and development of anticancer drugs. Br. J. Cancer *121*, 101–108.

54. André, T., Shiu, K.K., Kim, T.W., Jensen, B.V., Jensen, L.H., Punt, C., Smith, D., Garcia-Carbonero, R., Benavides, M., Gibbs, P., et al. (2020). Pembrolizumab in microsatellite-instability-high advanced colorectal cancer. N. Engl. J. Med. *383*, 2207–2218.

55. Marcus, L., Lemery, S.J., Keegan, P., and Pazdur, R. (2019). FDA approval summary: pembrolizumab for the treatment of microsatellite instability-high solid tumors. Clin. Cancer Res. *25*, 3753–3758.

YMTHE, Volume *31*

Supplemental Information

Inducing mismatch repair deficiency sensitizes immune-cold neuroblastoma to anti-CTLA4 and generates broad anti-tumor immune memory

Mikal El-Hajjar, Lara Gerhardt, Megan M Y Hong, Mithunah Krishnamoorthy, Rene Figueredo, Xiufen Zheng, James Koropatnick, and Saman Maleki Vareki

Supplementary Data

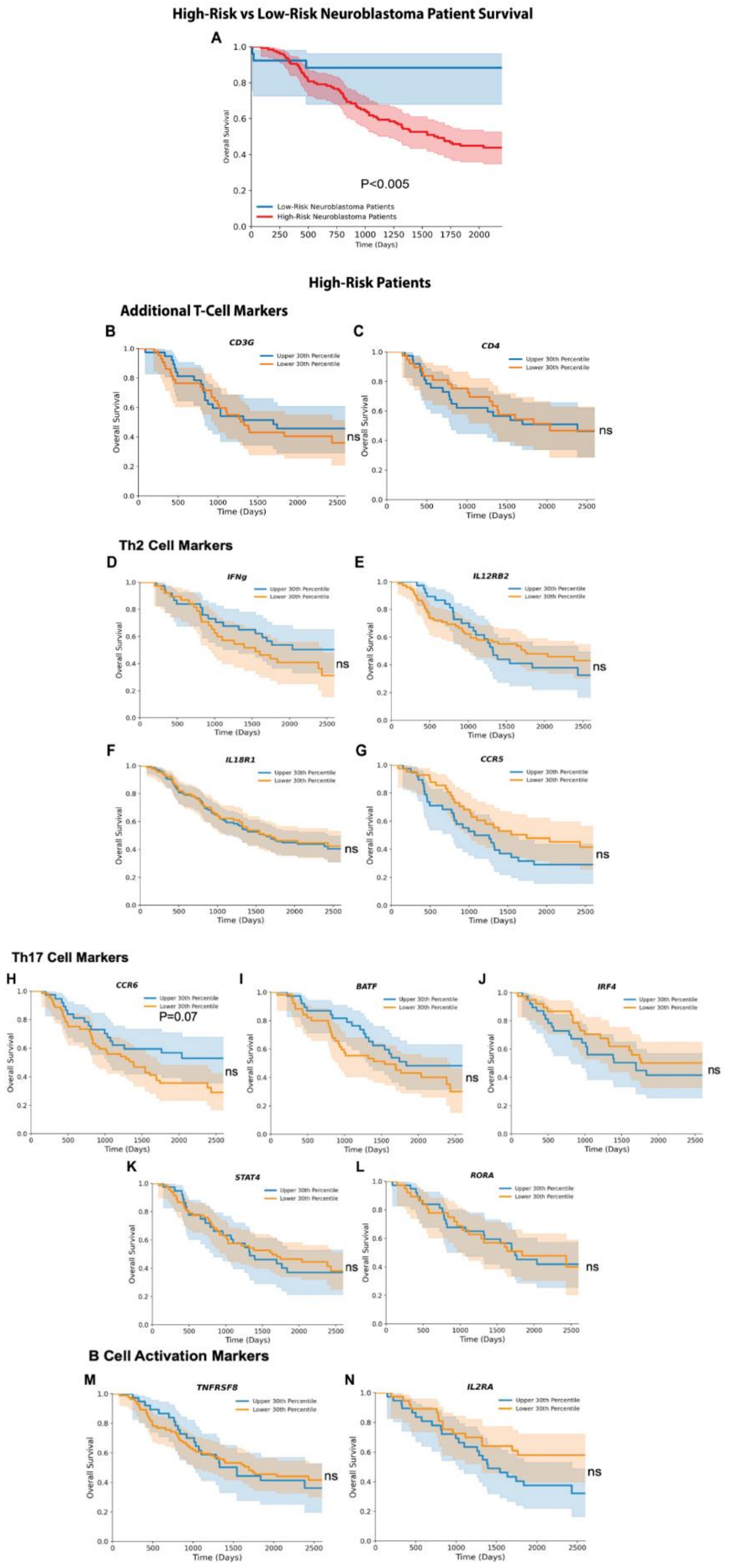

Figure S1: Th1, Th2, Th17 and B-cell gene expression are not associated with improved survival in high-risk neuroblastoma patients. Kaplan-Meier survival analysis by log-rank test of low-risk neuroblastoma patients (n=26) vs. high-risk neuroblastoma patients (n=127) (**A**). Kaplan-Meier survival analysis by log-rank test of high-risk neuroblastoma patients stratified according to upper and lower 30th percentile gene expression of CD3G (**B**) (n=38, n=43), CD4 (**C**) (n=38, n=38), IFNg (**D**) (n=38, n=38), IL12RB2 (**E**) (n=38, n=73), IL18R1 (**F**) (n=127, n=91), CCR5 (**G**) (n=38, n=42), CCR6 (**H**) (n=38, n=62), BATF (**I**) (n=38, n=52), IRF4 (**J**) (n=38, n=38), STAT4 (**K**) (n=38, n=67), RORA (**L**) (n=38, n=38), TNFRSF8 (**M**) (n=38, n=84), IL2RA (**N**) (n=38, n=38). * $p \leq 0.05$, ns (not significant).

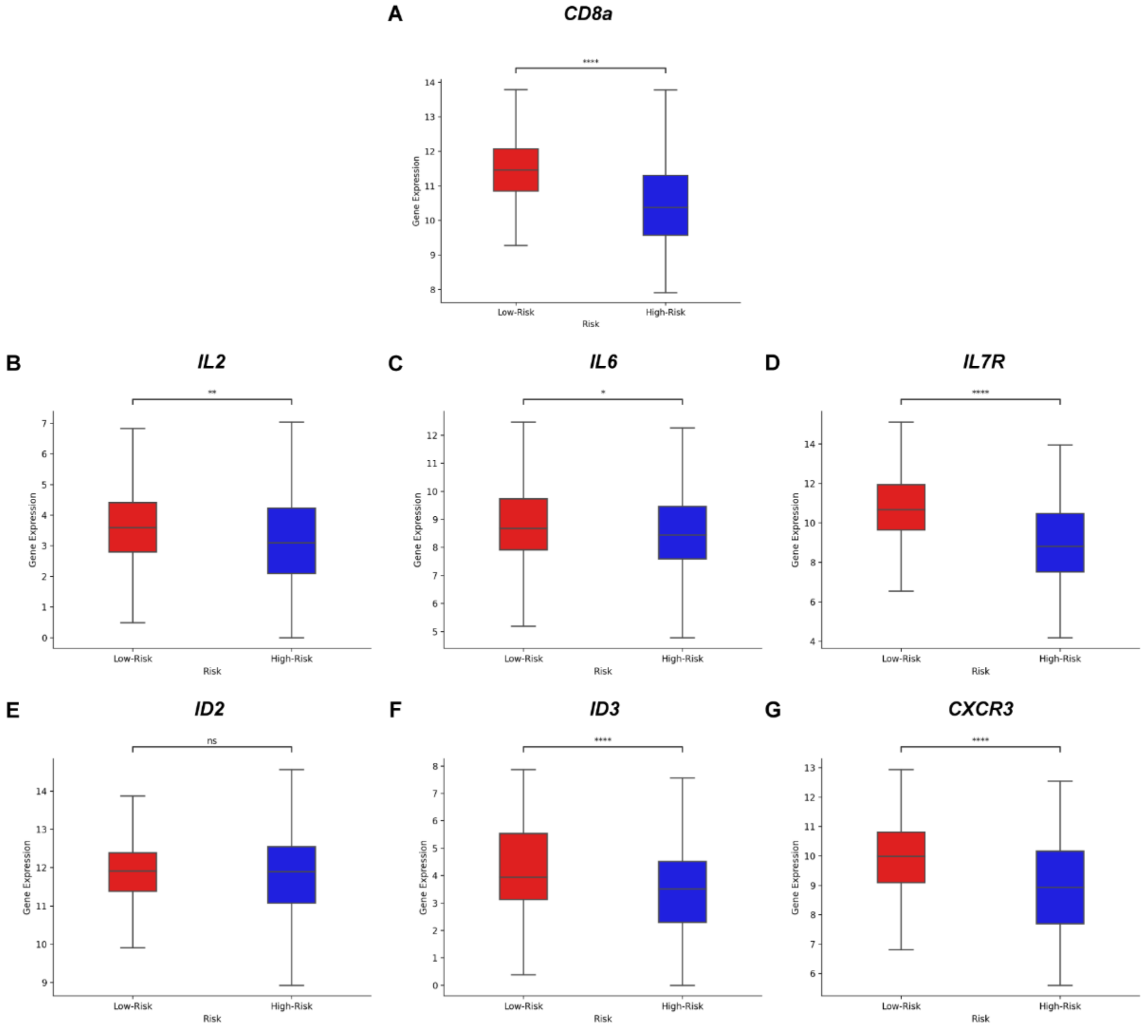

Figure S2: Low-risk neuroblastoma patients have an increased expression of pro-inflammatory genes compared to high-risk neuroblastoma patients. Tumor samples obtained from the neuroblastoma GEO49710 dataset were grouped into low-risk and high-risk subsets respectively (n=322, n=176). Normalized microarray gene expression reads for T-cell associated genes CD3a (**A**), IL2 (**B**), IL6 (**C**), IL7R (**D**), ID2 (**E**), ID3 (**F**) was compared. Statistical analysis was performed by non-parametric Mann-Whitney U test. * $p \leq 0.05$, ** $p \leq 0.01$, *** $p \leq 0.001$, **** $p \leq 0.0001$, ns (not significant).

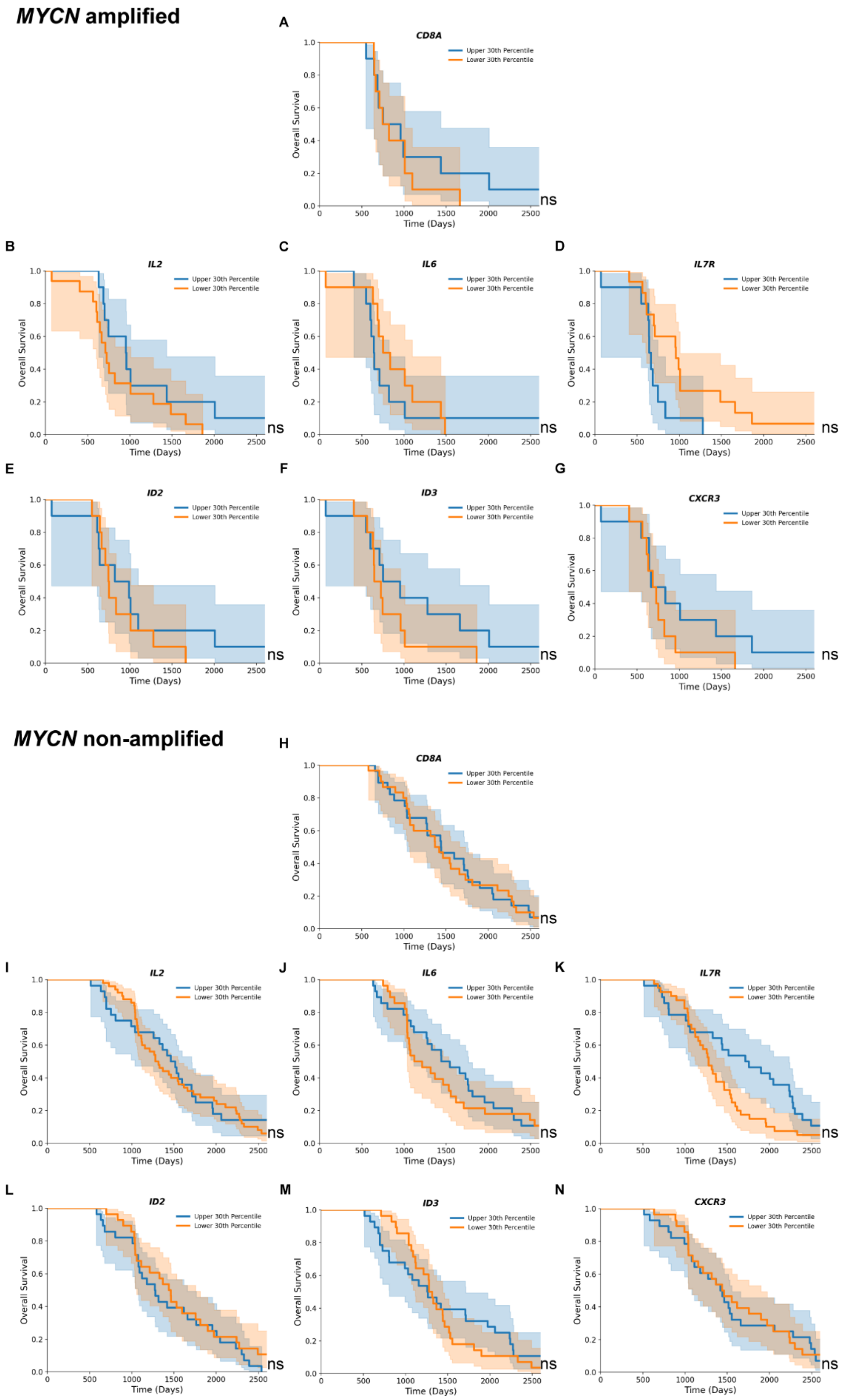

MYCN amplified
A
CD8A
B
IL2
C
IL6
D
IL7R
E
ID2
F
ID3
G
CXCR3
MYCN non-amplified
H
CD8A
I
IL2
J
IL6
K
IL7R
L
ID2
M
ID3
N
CXCR3
Upper 30th Percentile
Lower 30th Percentile
Overall Survival
Time (Days)
ns

Figure S3: Expression of proinflammatory genes are not correlated with *MYCN* amplification status in high-risk neuroblastoma patients. Kaplan-Meier survival analysis by log-rank test of neuroblastoma patients with *MYCN* amplification stratified according to upper and lower 30th percentile gene expression of CD8a (**A**) (n=10, n=10), IL2 (**B**) (n=10, n=16), IL6 (**C**) (n=10, n=10), IL7R (**D**) (n=10, n=15), ID2 (**E**) (n=10, n=10), ID3 (**F**) (n=10, n=10) and CXCR3 (**G**) (n=10, n=10). Similar analysis was conducted in non-amplified neuroblastoma patients, stratifying patients according to expression of genes CD8a (**H**) (n=28, n=30), IL2 (**I**) (n=28, n=50), IL6 (**J**) (n=28, n=28), IL7R (**K**) (n=28, n=40), ID2 (**L**) (n=28, n=28), ID3 (**M**) (n=28, n=28) and CXCR3 (**N**) (n=28, n=28). ns (not significant)

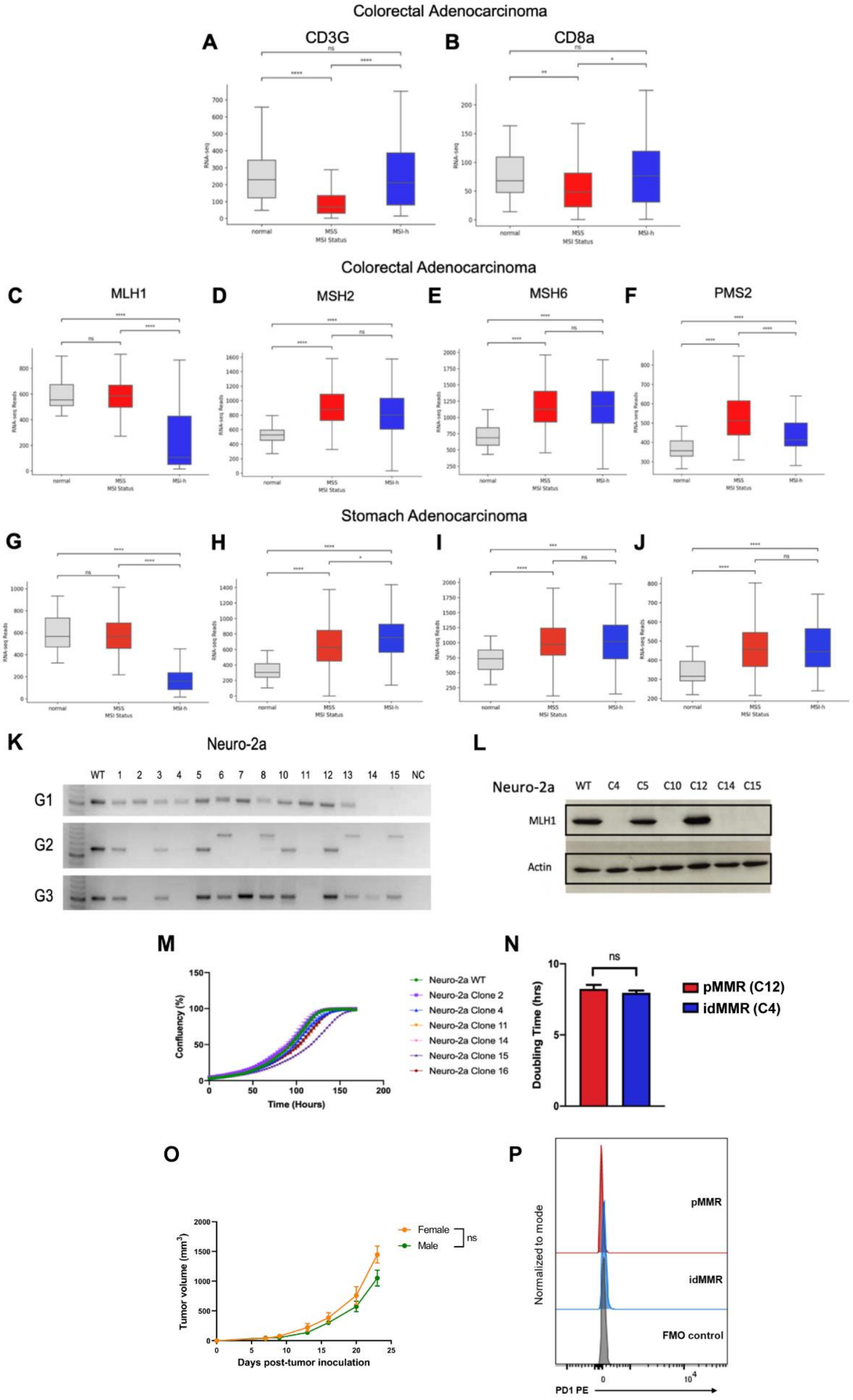

Colorectal Adenocarcinoma
A
CD3G
B
CD8a
RNA-seq
normal
MSS
MSI-h
MSI Status
Colorectal Adenocarcinoma
C
MLH1
D
MSH2
E
MSH6
F
PMS2
RNA-seq Reads
Stomach Adenocarcinoma
G
H
I
J
K
Neuro-2a
WT
NC
G1
G2
G3
L
Neuro-2a
WT
C4
C5
C10
C12
C14
C15
MLH1
Actin
M
Confluency (%)
Time (Hours)
Neuro-2a WT
Neuro-2a Clone 2
Neuro-2a Clone 4
Neuro-2a Clone 11
Neuro-2a Clone 14
Neuro-2a Clone 15
Neuro-2a Clone 16
N
ns
Doubling Time (hrs)
pMMR (C12)
idMMR (C4)
O
Tumor volume (mm^3)
Days post-tumor inoculation
Female
Male
ns
P
Normalized to mode
pMMR
idMMR
FMO control
PD1 PE

Figure S4: MMR deficiency enhances T-cell based immune surveillance in tumors. Normalized RNA-seq data for T-cell genes *CD3G* (**A**), *CD8A* (**B**), *MLH1* (**C**), *MSH2* (**D**), *MSH6* (**E**), *PMS2* (**F**) from the TCGA database colorectal adenocarcinoma (COAD READ) dataset and *MLH1* (**G**), *MSH2* (**H**), *MSH6* (**I**), *PMS2* (**J**) from the stomach adenocarcinoma (STAD) dataset. COAD READ and STAD samples were grouped into normal, MSS and MSI-h tissues (n=51, n=257, n=51), (n=35, n=276, n=80) respectively. Statistical analysis was performed by non-parametric Mann-Whitney U test. * $p \leq 0.05$, ** $p \leq 0.01$, *** $p \leq 0.001$, **** $p \leq 0.0001$, ns (not significant). Neuro-2a cells were transfected with *MLH1* CRISPR CAS9 knockout plasmid. PCR results for clones resulted from transfection of neuro-2a cells with *MLH1* CRISPR/Cas9 plasmid (top to bottom: amplification with guide 1 (G1), G2 and G3 primers respectively), WT represents amplification of non-transfected wild type (WT) Neuro-2a cells as a positive control for each primer and NC represents a negative control for each set of PCR products (no template) (**K**). Expression of MLH1 in the parental cells and several putative knockout clones was analyzed by Western Blot(**L**). WT neuro-2a and *MLH1* KO neuro-2a cell growth profiles over a 90-hour period imaged using Incucyte (**M**). WT neuro-2a cells (pMMR) (n=9) and *MLH1* KO neuro-2a cells (idMMR) (n=9) were seeded on day 0 and counted on day 1 and were counted again on day 5 to analyze the fold increase of each neuro-2a clone (**N**). Results are representative of 3 pooled experiments. Comparison of idMMR tumor growth in immunocompetent A/J male and female mice (n=5, n=5) (**O**). Flow cytometric analysis of PD1 expression on 8-week pMMR and idMMR neuro-2a cells (**N**). Statistical analysis was performed by unpaired two-tailed Student's t-test, ns (not significant).

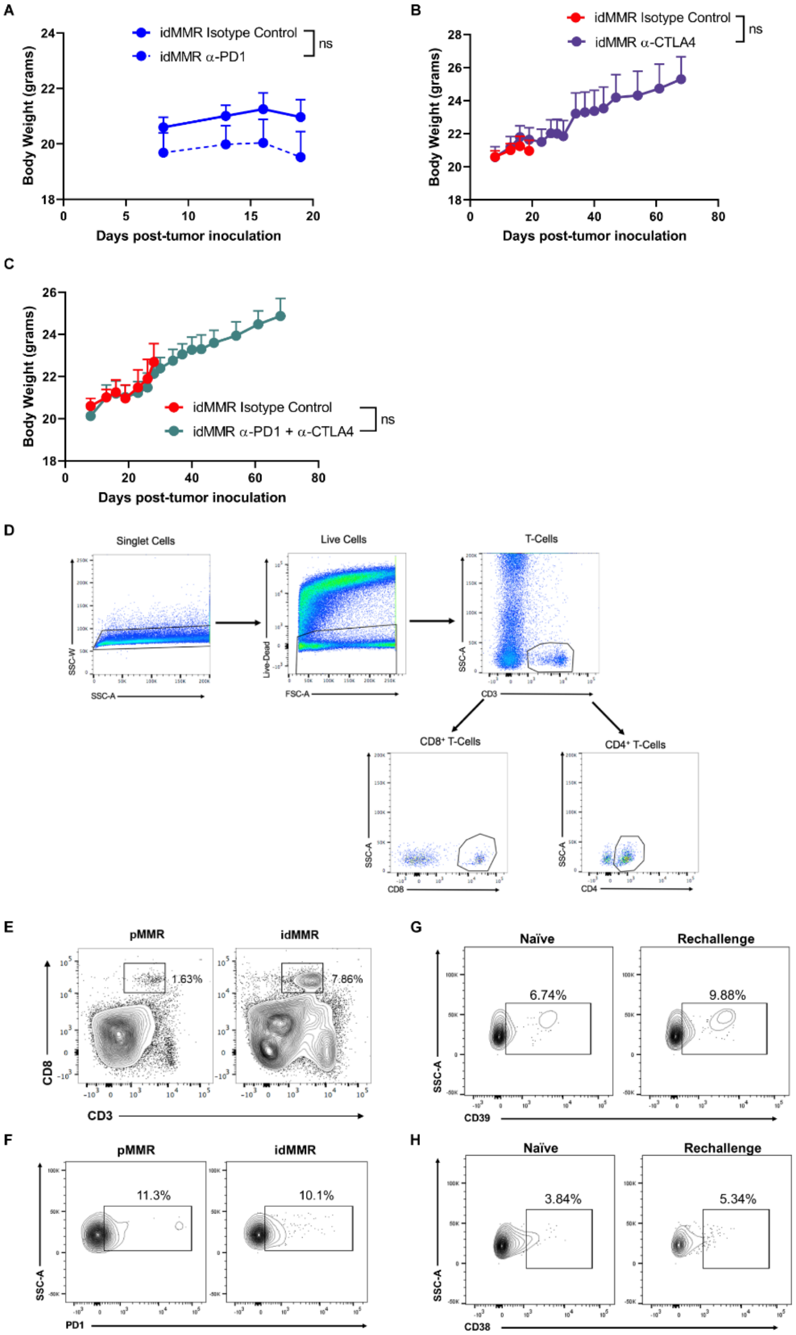

Figure S5: Weight of mice throughout anti-PD1 and anti-CTLA4 treatment. Animals were treated with isotype control antibodies or anti-PD1 antibody (**A**), anti-CTLA4 (**B**), or combination of anti-PD1 and anti-CTLA4 antibodies and their body weight was measured overtime (**C**). Representative flow cytometry gating strategy. Singlet cells were gated and doublet and triplet are excluded. Dead cells were excluded using Zombie NIR live-dead dye. Live T-cells were gated using CD3 marker. CD8 and CD4 T-cells were gated from the T-cell population using CD8 and CD4 markers. Gates were determined using a fluorescence minus one (FMO) control (**D**). Representative plots of $CD3^+CD8^+$ (**E**) and $PD1^+$ $CD8^+$ T-cells (**F**). Representative plots of $CD39^+$ $CD8^+$ T-cells (**G**) and $CD38^+$ $CD4^+$ T-cells (**H**).

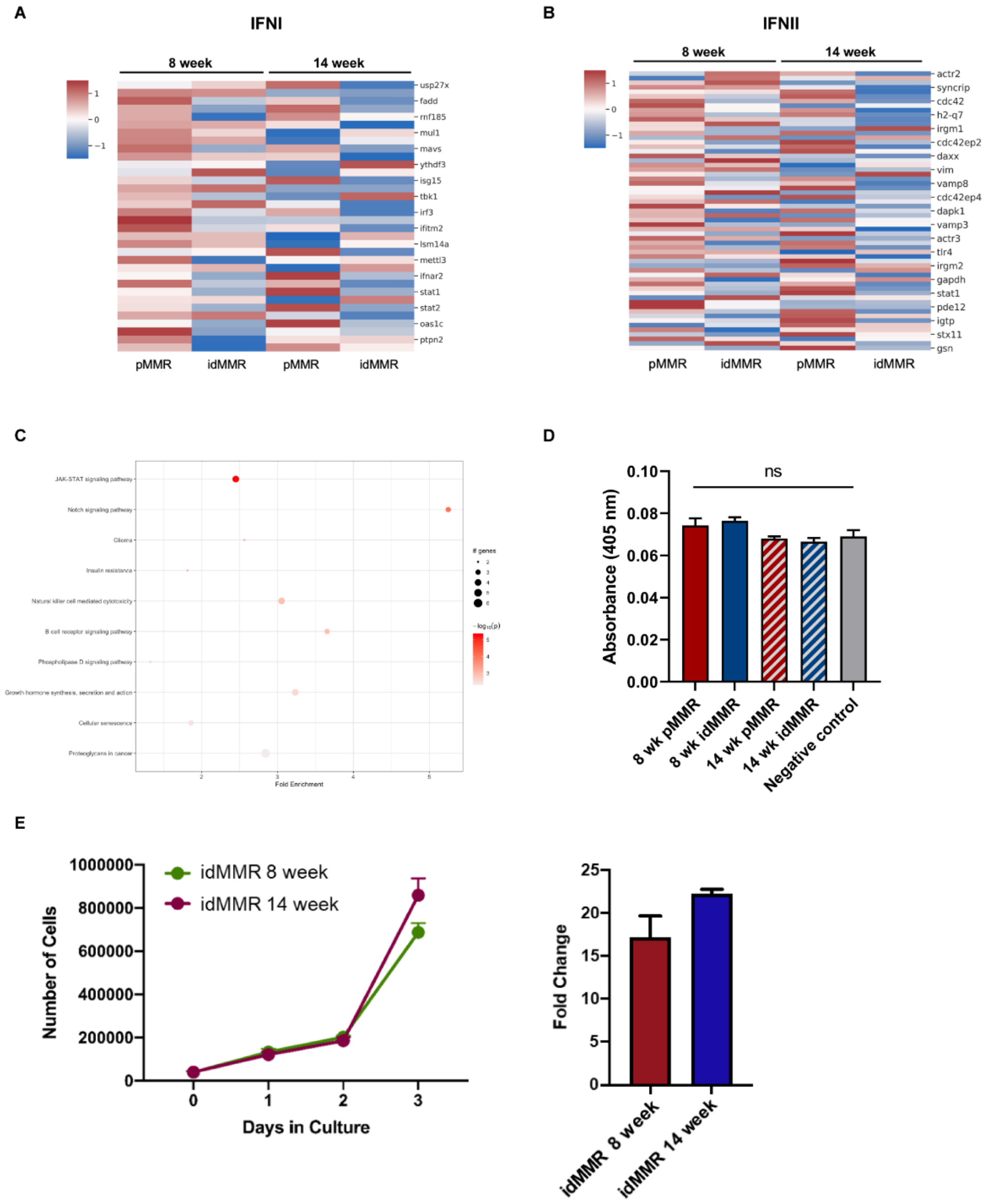

Figure S6: IFN-related gene expression of pMMR and idMMR neuroblastoma cells at 8 weeks and 14 weeks of culture and growth kinetics. Heat maps of the expression of IFNI- and IFNII-related genes in pMMR and idMMR neuroblastoma cells (**A**, **B**). Pathway enrichment analysis of downregulated pathways in idMMR cells compared to pMMR cells at 8 weeks (**C**). IFN-β production from cell culture supernatant measured by ELISA (**D**). Comparison of growth kinetics of idMMR neuroblastoma cells at 8 and 14 weeks of culture. 8-week and 14-week idMMR cells were seeded on day 0 and counted on day 1 and 3 to analyze the cell growth over time fold. Fold increase of both cell lines grown was calculated using cell counts on day 1 and 3 (**E**).

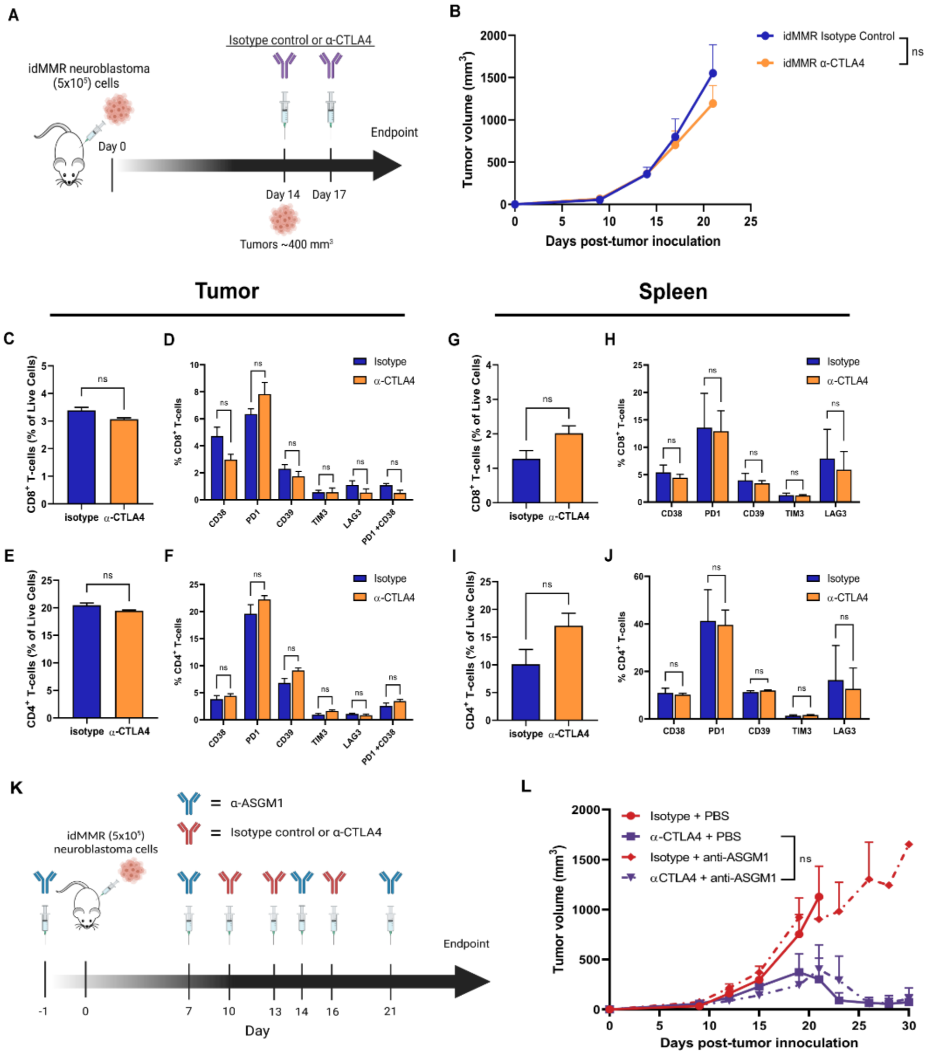

Figure S7: Anti-CTLA4 treatment does not induce inhibitory molecules on T-cells in a failure setting and the effect of anti-CTLA4 against idMMR neuroblastoma tumors is not NK cell dependent. On day 0, mice were injected subcutaneously with idMMR neuro-2a cells (500,000), then on days 14 and 17, they were either administered the isotype control or anti-CTLA4 antibody (n=3, n=5, respectively) (**A**). Tumor growth (**B**). On day 21, tumors and spleens were harvested and processed for analyzing TILs (left panel) and T-cells (right panel) using flow cytometry. Left panel, graphs are presented as a percentage of live cells, CD3$^+$CD8$^+$ TILs (**C**) or CD3$^+$CD4$^+$ TILs (**E**). Quantification of CD38$^+$, PD1$^+$, CD39$^+$, TIM3$^+$, LAG3$^+$, and PD1$^+$CD38$^+$ cells as a percentage of CD8$^+$ (**D**) and CD4$^+$ (**F**) T-cells from staining tumor samples. Data represent mean of technical replicates. Right panel, graphs are presented as a percentage of live cells, CD3$^+$CD8$^+$ T-cells (**G**) or CD3$^+$CD4$^+$ T-cells (**I**). Quantification of CD38$^+$, PD1$^+$, CD39$^+$, TIM3$^+$, LAG3$^+$ cells as a percentage of CD8$^+$ (**H**) and CD4$^+$ (**J**) T-cells from stained splenocyte samples. Experimental design and tumor growth of idMMR neuro-2a-bearing A/J mice treated with isotype control, anti-asialo GM1 (α-ASGM1) for NK cell depletion, anti-CTLA4, or anti-CTLA4 with α-ASGM1 (**K, L**). Statistical analyses were performed using the unpaired two-tailed Student's t-test or two-way ANOVA with Sidak's multiple comparisons test, ns (not significant), error bars indicate SEM.
